# PBPK Model of Coproporphyrin I: Evaluation of the Impact of SLCO1B1 Genotype, Ethnicity, and Sex on its Inter‐Individual Variability

**DOI:** 10.1002/psp4.12582

**Published:** 2021-01-19

**Authors:** Hiroyuki Takita, Shelby Barnett, Yueping Zhang, Karelle Ménochet, Hong Shen, Kayode Ogungbenro, Aleksandra Galetin

**Affiliations:** ^1^ Centre for Applied Pharmacokinetic Research Division of Pharmacy and Optometry School of Health Sciences Faculty of Biology, Medicine and Health University of Manchester Manchester UK; ^2^ Laboratory for Safety Assessment and ADME Pharmaceuticals Research Center Asahi Kasei Pharma Corporation Shizuoka Japan; ^3^ Pharmaceutical Candidate Optimization Bristol-Myers Squibb Princeton New Jersey USA; ^4^ Quantitative Pharmacology & DMPK UCB Slough UK

## Abstract

Coproporphyrin I (CPI) is an endogenous biomarker of OATP1B activity and associated drug‐drug interactions. In this study, a minimal physiologically‐based pharmacokinetic model was developed to investigate the impact of OATP1B1 genotype (c.521T>C), ethnicity, and sex on CPI pharmacokinetics and interindividual variability in its baseline. The model implemented mechanistic descriptions of CPI hepatic transport between liver blood and liver tissue and renal excretion. Key model parameters (e.g., endogenous CPI synthesis rate, and CPI hepatic uptake clearance) were estimated by fitting the model simultaneously to three independent CPI clinical datasets (plasma and urine data) obtained from white (*n* = 16, men and women) and Asian‐Indian (*n* = 26, all men) subjects, with c.521 variants (TT, TC, and CC). The optimized CPI model successfully described the observed data using c.521T>C genotype, ethnicity, and sex as covariates. CPI hepatic active was 79% lower in 521CC relative to the wild type and 42% lower in Asian‐Indians relative to white subjects, whereas CPI synthesis was 23% higher in male relative to female subjects. Parameter sensitivity analysis showed marginal impact of the assumption of CPI synthesis site (blood or liver), resulting in comparable recovery of plasma and urine CPI data. Lower magnitude of CPI‐drug interaction was simulated in 521CC subjects, suggesting the risk of underestimation of CPI‐drug interaction without prior OATP1B1 genotyping. The CPI model incorporates key covariates contributing to interindividual variability in its baseline and highlights the utility of the CPI modeling to facilitate the design of prospective clinical studies to maximize the sensitivity of this biomarker.

**STUDY HIGHLIGHTS**

**WHAT IS THE CURRENT KNOWLEDGE ON THE TOPIC?**

Reduced activity of OATP1B1 in homozygous carriers of c.521T>C resulted in higher plasma concentration of coproporphyrin I (CPI), an endogenous biomarker of OATP1B. Women showed lower CPI plasma concentrations than men.

**WHAT QUESTION DID THIS STUDY ADDRESS?**

To what extent do OATP1B1 c.521T>C, ethnicity, and sex affect CPI steady‐state baseline and the extent of CPI‐drug interaction? How does the assumption of CPI synthesis site affect model performance?

**WHAT DOES THIS STUDY ADD TO OUR KNOWLEDGE?**

Modeling of CPI identified three covariates; decreased hepatic CPI uptake in 521CC relative to 521TT, Asian‐Indians relative to white subjects, and lower CPI synthesis in women relative to men. These results were mostly unaffected by the assumption on CPI synthesis site in the model. Theoretical simulation evaluated the impact of each covariate on CPI‐drug interaction risk.

**HOW MIGHT THIS CHANGE DRUG DISCOVERY, DEVELOPMENT, AND/OR THERAPEUTICS?**

Modeling of CPI maximizes the sensitivity of this biomarker to evaluate OATP1B1 interaction potential as early as in first‐in‐human studies and to facilitate the design of prospective interaction studies with corresponding clinical probes.

Hepatic uptake via OATP1B1 and 1B3 has been widely recognized as a rate‐limiting step in the clearance of anionic drugs, such as statins.^1^, ^2^ Considering the high degree of drug‐drug interactions (DDIs) associated with OATP1B1 and therapeutic implications, regulatory agencies suggest elucidating factors that can change pharmacokinetics (PKs) of OATP1B1 substrates (https://www.fda.gov/media/134581/download, https://www.ema.europa.eu/en/documents/scientific‐guideline/guideline‐investigation‐drug‐interactions‐revision‐1_en.pdf). Genetic polymorphism of *SLCO1B1* coding OATP1B1, is one of the factors affecting exposure of OATP1B1 substrates.^3^, ^4^ Subjects with c.521T>C mutation (521CC), associated with reduced transporter activity, have higher exposure of OATP1B substrates relative to wild‐type allele (521TT).^5^ In addition, 521CC subjects showed lower fold‐change in the area under the plasma concentration‐time curve ratio (AUCR) of statins in the presence of OATP1B inhibitors,^3^, ^6^ likely due to reduced contribution of OATP1B1 to the elimination in these individuals. In addition, several studies reported increased exposure of statins in Asians (mainly Japanese) relative to whites,^4^, ^7^, ^8^, ^9^ resulting in the reduced starting dose of OATP1B substrates (e.g., rosuvastatin) in this population.^10^ Increased exposure of statins in Japanese was attributed to the intrinsic ethnic variability in OATP1B activity, in addition to genetic polymoprhism,^4^, ^11^ although some studies have challenged this hypothesis.^12^, ^13^ Recently, application of this assumption in letermovir physiologically‐based PK (PBPK) modeling explained ethnic difference in the PKs of this drug.^14^

Recent years have seen an increased application of endogenous biomarkers as novel tools to investigate transporter function *in vivo*, with the primary aim to de‐risk transporter‐mediated DDIs in early drug development.^1^, ^15^, ^16^, ^17^ This trend has been particularly evident for OATP1B. Coproporphyrin I (CPI), a byproduct of heme synthesis, is one of the most promising endogenous biomarkers of OATP1B. It is a metabolically stable substrate of OATP1B1 and 1B3, as well as MRP2 and MRP3 transporters.^18^, ^19^, ^20^, ^21^, ^22^, ^23^ CPI‐drug interactions reported so far in human^22^, ^24^, ^25^, ^26^, ^27^, ^28^ and preclinical species^19^, ^29^, ^30^ suggest that this biomarker is expected to closely follow the perpetrator concentration–time profile. In contrast to other OATP1B biomarkers, circadian rhythm or food intake are not likely to cause interindividual variability in its plasma baseline.^19^, ^24^

Several endogenous factors can affect the disposition of CPI. Hepatic uptake via OATP1B is the main elimination route of CPI (> 85%)^18^ and increased CPI baseline was recently reported in OATP1B1 521CC,^25^, ^31^ whereas other genetic mutations of OATP1B (e.g., c.388A>G) showed no effect.^28^, ^31^ Higher proportion of CPI in urine in patients with MRP2 mutations (Dubin–Johnson syndrome)^32^ were reported, with no strong evidence of altered plasma CPI concentrations. Ethnic differences in baseline CPI were not highlighted in previous studies including white and Asian subjects.^15^, ^28^ However, these studies did not consider OATP1B1 genotype in conjunction with ethnicity as a covariate. Changes in CPI synthesis may also alter CPI baseline. Increased urinary and fecal excretion of CPI was observed when hemogenesis is triggered by anemia^33^ or hemolysis.^34^ In addition, lower CPI baseline was reported in Japanese female subjects relative to male subjects, with no sex‐related differences in exposure of other OATP1B markers or probes, suggesting reduced CPI synthesis in women.^31^

Several groups, including us, have reported CPI models with different complexities, ranging from a turnover model to more complex description of the processes in the liver, developed with the aim to facilitate quantitative understanding of CPI disposition.^18^, ^35^, ^36^ In all modeling examples, hepatic and nonhepatic routes were considered, but there were inconsistencies in terms of CPI synthesis site; it was assumed to occur either in the blood (central) compartment^18^, ^35^ or liver,^36^ with no clear consensus.

This study aimed to evaluate quantitatively the impact of genetic polymorphism of *SLCO1B1* c.521T>C, ethnicity, and sex on interindividual variability in CPI baseline using the largest clinical dataset so far. To estimate the effect of these covariates and to account for the mechanistic description of hepatic transporter processes, the CPI model was extended from our previous work.^18^ Parameter estimation was implemented by fitting the model simultaneously to three independent CPI clinical datasets, including white (*n* = 16, men and women) and Asian‐Indian (*n* = 26, neb) subjects and included all *SLCO1B1* c.521T>C variants (521TT, TC, and CC). Comprehensive literature search on the available biological information related to CPI synthesis was performed and the impact of different assumptions of the CPI synthesis site was assessed by parameter sensitivity analysis using the extended CPI model. Finally, the impact of the covariates on CPI‐drug OATP1B‐mediated interaction potential was simulated to support optimal design of prospective clinical studies.

## METHODS

### Individual CPI clinical data

Individual CPI data were obtained from three clinical studies (**Table** **1**). Shen *et al*.^37^ (study 1) was conducted in 14 healthy male Asian‐Indians (521TT; *n* = 13, 521TC; *n* = 1) and CPI baseline plasma concentrations were measured over 24 hours (**Supplementary Material Section** **S1**). Yee *et al*.^25^ (study 2) was conducted in 16 healthy Caucasians (521TT; *n* = 8, 521TC; *n* = 6, and 521CC; *n* = 2); only CPI concentration in plasma was measured on 1 occasion over 12 hours. Lai *et al*.^24^ (study 3) was conducted in 12 healthy male Asian‐Indians (all 521TT) subjects and CPI concentrations in plasma and urine were measured over 24 hours on 3 occasions; 2 occasions involved interaction with rifampicin. Data on three main covariates (*SLCO1B1* c.521T>C status, ethnicity, and sex) investigated here were available for all 42 subjects.

**Table 1:** Clinical data used in CPI population PBPK model development

No. subjects	Study 1 (Shen *et al*. 2019)^37^	Study 2 (Yee *et al*. 2019)^25^		Study 3 (Lai *et al*. 2019)^24^
	14	8	8	12
Ethnicity	Asian‐Indians	Caucasian	Caucasian	Asian‐Indians
Sex	Male	Male	Female	Male
Study duration	24h	12h	12h	24h
SLCO1B1 c.521 Genotype	TT (*n* = 13), TC (*n* = 1)	TT (*n* = 4), TC (*n* = 3), CC (*n* = 1)	TT (*n* = 4), TC (*n* = 3), CC (*n* = 1)	TT (*n* = 12)
No. occasions; treatment	Occ 1; Predose Occ 2; Furosemide	Occ 1; Pravastatin	Occ 1; Pravastatin	Occ 1; Rifampicin, Occ 2; Rosuvastatin Occ 3; Rifampicin + Rosuvastatin
No. plasma samples	Occ 1; 155 (TT) and 12 (TC) Occ 2; 151 (TT) and 11 (TC)	(TT); 58 (TC); 44 (CC); 15	(TT); 57 (TC); 41 (CC); 14	Occ 1; 144 Occ 2; 132 Occ 3; 132
No. urine samples	0	0	0	34 (pre‐treatment) 68 (post‐treatment)
C_ave,base_, nM	TT; 1.07 ± 0.26 TC; 1.86	TT; 0.66 ± 0.11 TC; 1.08 ± 0.14 CC; 1.74	TT; 0.54 ± 0.05 TC; 0.69 ± 0.13 CC; 1.37	TT; 0.87 ± 0.16

C_ave,base_, coproporphyrin I concentration in plasma over whole study period in occasions without rifampicin treatment; CPI, coproporphyrin I; Occ, occasion.

### CPI population PBPK model

**Figure 1:**
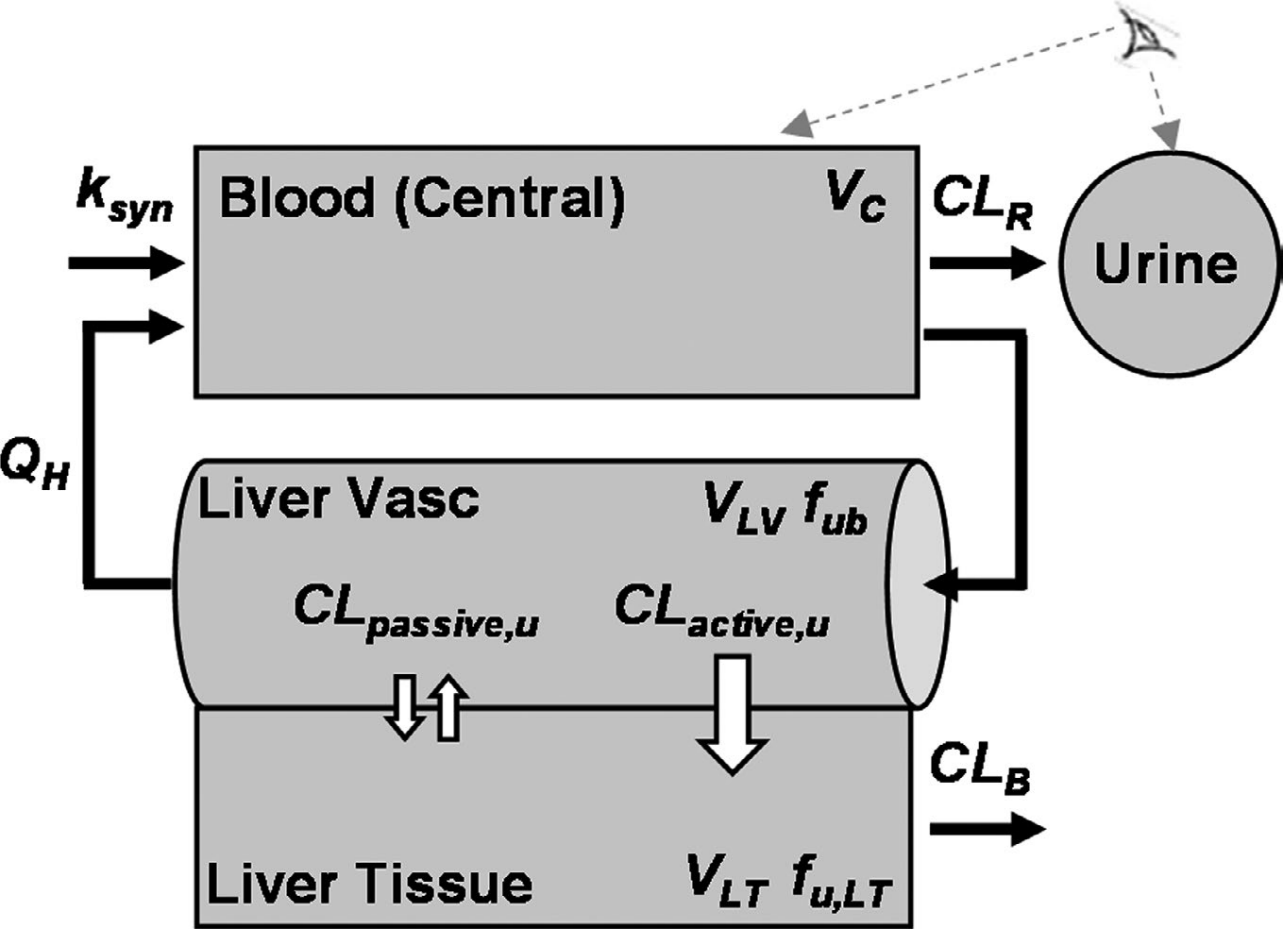
Structure of the coproporphyrin I minimal PBPK model. *CL_active,u_*, hepatic uptake clearance (unbound); *CL_B_*, biliary clearance; *CL_passive,u_*, hepatic passive clearance (unbound); *CL_R_*, renal clearance; *f_ub_*, fraction unbound in the blood; *f_u,LT_*, fraction unbound in liver tissue; *k_syn_*, endogenous synthesis rate; *Q_H_*, hepatic blood flow; *V_C_*, volume of central compartment; *V_LV_*, volume of liver vascular; *V_LT_*, volume of liver tissue. An eye symbol represents observed compartments.

The CPI model structure was based on our CPI model published previously,^18^ but included a mechanistic description of the liver compartment (**Figure** **1**). The model consists of five compartments, including blood (central), urine, liver vascular, liver tissue, and bile compartments.^38^ The ordinary differential equations describing the concentration/amount in these compartments are:

(1) $$\frac{dC_{\mathrm{blood}}}{\mathrm{dt}}=\left(k_{\mathrm{syn}}‐CL_{R}\cdot C_{\mathrm{blood}}+Q_{H}\cdot \left(C_{\mathrm{LV}}‐C_{\mathrm{blood}}\right)\right)\cdot \frac{1}{V_{c}}$$

(2) $$\frac{dC_{\mathrm{LV}}}{\mathrm{dt}}=\left(\begin{array}{c} Q_{H}\cdot \left(C_{\mathrm{blood}}‐C_{\mathrm{LV}}\right)+CL_{passive,u}\cdot \left(\left(C_{\mathrm{LT}}\cdot fu_{\mathrm{LT}}\right)‐\left(C_{\mathrm{LV}}\cdot fu_{b}\right)\right)\\ ‐CL_{active,u}\cdot C_{\mathrm{LV}}\cdot fu_{b} \end{array}\right)\cdot \frac{1}{V_{\mathrm{LV}}}$$

(3) $$\frac{dC_{\mathrm{LT}}}{\mathrm{dt}}=\left(\begin{array}{c} CL_{passive,u}\cdot \left(\left(C_{\mathrm{LV}}\cdot fu_{b}\right)‐\left(C_{\mathrm{LT}}\cdot fu_{\mathrm{LT}}\right)\right)+CL_{active,u}\cdot C_{\mathrm{LV}}\cdot fu_{b}\\ ‐CL_{B}\cdot C_{\mathrm{LT}}\cdot fu_{\mathrm{LT}} \end{array}\right)\cdot \frac{1}{V_{\mathrm{LT}}}$$

(4) $$\frac{dA_{\mathrm{bile}}}{\mathrm{dt}}=CL_{B}\cdot C_{\mathrm{LT}}\cdot fu_{\mathrm{LT}}$$

(5) $$\frac{dA_{\mathrm{urine}}}{\mathrm{dt}}=CL_{R}\cdot C_{\mathrm{blood}}$$

where *C_blood_*, *C_LV_*, *C_LT_*, *A_bile_*, and *A_urine_* are the concentration (*C*) or amount (*A*) of CPI in the blood (central), liver vascular, liver tissue, bile, and urine compartments, respectively. The parameters *k_syn_*, *Q_H_*, *V_c_*, *V_LV_*, *V_LT_*, *CL_R_*, *CL_passive,u_*, *CL_active,u_*, *CL_B_*, *fu_b_*, and *fu_LT_* are rate of CPI synthesis, hepatic blood flow, volume of blood (central) compartment, volume of liver vascular, volume of liver tissue, renal clearance, hepatic passive clearance (unbound), hepatic active uptake clearance (unbound), biliary clearance, fraction unbound in blood, and faction unbound in liver tissue, respectively. Due to lack of corroborating evidence of CPI enterohepatic recirculation^29^ and low bioavailability of labeled CPI in monkeys,^30^ this process was assumed to be insignificant for the PK of CPI and was not considered in the current model development.

Competitive inhibition of OATP1B‐mediated *CL_active,u_* by rifampicin was described by Eqs. 6 and 7.

(6) $$\frac{dC_{\mathrm{LV}}}{\mathrm{dt}}=\left(Q_{H}\cdot \left(C_{\mathrm{blood}}‐C_{\mathrm{LV}}\right)+CL_{passive,u}\cdot \left(\left(C_{\mathrm{LT}}\cdot fu_{\mathrm{LT}}\right)‐\left(C_{\mathrm{LV}}\cdot fu_{b}\right)\right)‐\left(\frac{CL_{active,u}\cdot C_{\mathrm{LV}}\cdot fu_{b}}{1+\frac{C_{\mathrm{Rif}}}{\mathrm{Ki}}}\right)\right)\cdot \frac{1}{V_{\mathrm{LV}}}$$

(7) $$\frac{dC_{\mathrm{LT}}}{\mathrm{dt}}=\left(CL_{passive,u}\cdot \left(\left(C_{\mathrm{LV}}\cdot fu_{b}\right)‐\left(C_{\mathrm{LT}}\cdot fu_{\mathrm{LT}}\right)\right)+\left(\frac{CL_{active,u}\cdot C_{\mathrm{LV}}\cdot fu_{b}}{1+\frac{C_{\mathrm{Rif}}}{\mathrm{Ki}}}\right)‐CL_{B}\cdot C_{\mathrm{LT}}\cdot fu_{\mathrm{LT}}\right)\cdot \frac{1}{V_{\mathrm{LT}}}$$

where *C_Rif_* is total rifampicin plasma concentration and *Ki* is the inhibition constant for OATP1B‐mediated *CL_active,u_*. *C_Rif_* was predicted using individual parameter estimates (*posthoc*) obtained during development of a population PK model for rifampicin, as reported previously.^18^

### Parameter estimation and covariate analysis

To improve the parameter identifiability, some CPI model parameters were fixed to *in vitro* determined or physiological values, namely *in vitro CL_passive,u_* was scaled to *in vivo CL_passive,u_* using hepatocellularity of 120 × 10^6^ cells/g of liver,^38^ and *f_u,LT_* was estimated from *in vitro* CPI uptake studies in human hepatocytes (**Supplementary Material Section** **S2**). *f_u,b_* was calculated from experimentally determined fraction unbound in plasma (*f_u,p_*), with labeled CPI measured in this study (**Supplementary Material Section** **S2**), and reported blood‐to‐plasma ratio (**Table** **S4**). *Q_H_* and volumes of compartments (with the exception of the central compartment) were fixed to the physiological values reported previously.^38^

Remaining model parameters were estimated using mixed‐effects modeling in NONMEM version 7.42 using ADVAN13 and first‐order conditional estimation with interaction method. The CPI model was fitted to all individual plasma and urine data simultaneously using combined proportional and additive residual error models for the residual unexplained variabilities of both plasma and urine data; simulated CPI blood concentrations were converted to plasma concentrations using blood‐to‐plasma ratio. Between subject variability (BSV) was estimated for *k_syn_*, *CL_B_*, *CL_R_*, and *V_c_* assuming log‐normal distribution of the parameters; between occasion variability (BOV) was estimated only for *CL_B_* because inclusion of BOV for other parameters did not improve model performance.

Three covariates were investigated in the CPI model using log‐likelihood ratio tests at a significance level of *p* < 0.05: the effects of *SLCO1B1* c.521T>C and/or ethnicity on *CL_active,u_*, and the effect of sex on *k_syn_*. *CL_active,u_* in 521TT subjects was represented as the reference value (*CL_active,0_*), and the fraction change for polymorphic TC and CC allelic variants (*COV_GEN_*) were represented as follows:

(8) $$CL_{active,u}=CL_{active,0}\cdot \left(1‐GEN\cdot COV_{\mathrm{GEN}}\right)$$

where *GEN* is a dummy variable that takes the following values: *GEN* = 0 (521TT), 0.5 (521TC), and 1 (521CC). Here, the fractional change in 521TC was assumed to be half of the value for 521CC (an additive genetic effect). To keep *COV_GEN_* between 0 and 1, *COV_GEN_* was estimated indirectly using a surrogate variable of genetic effect (*FRAX*) shown in Eq. 9:

(9) $$COV_{\mathrm{GEN}}=1/\left(1+FRAX\right)$$

where *FRAX* can have any positive real value.

The effect of ethnicity on *CL_active,u_* was also introduced as follows:

(10) $$\begin{array}{cc} CL_{active,u}= & CL_{active,0}\cdot \left(1‐RACE\cdot COV_{\mathrm{RACE}}\right)\\ & \cdot \left(1‐GEN\cdot COV_{\mathrm{GEN}}\right) \end{array}$$

where *CL_active,0_* is for Caucian subjects with 521TT genotype (reference group), *COV_RACE_* is the fractional change in *CL_active,u_* in Asian‐Indians relative to Caucasians, and *RACE* is a dummy variable that takes the value of 0 for Caucians and 1 for Asian‐Indians.

The effect of sex on CPI synthesis rate (*k_syn,sex_*) was described as follows:

(11) $$k_{syn,sex}=k_{syn,male}\cdot \left(1‐SEX\cdot COV_{\mathrm{SEX}}\right)$$

where *COV_SEX_* is the fractional change in *k_syn_* in women relative to men, and *SEX* is a dummy variable that takes the value of 0 for men and 1 for women.

Data exploration and goodness‐of‐fit (GOF) plots were produced using MATLAB (R2017a version 9.2.0). GOF plots, such as observed data vs. population predicted (PRED) or individual PRED, conditionally weighted residual vs. TIME or population prediction were used to assess possible model misspecification. Visual predictive check (VPC) based on 5,000 simulated individuals using the covariate demographics (*SLCO1B1* c.521T>C, ethnicity, and sex) was used to assess final model performance.

### Location of CPI synthesis

The plausibility of the conflicting hypotheses on the site of the CPI synthesis (blood or liver) was assessed by comprehensive literature search. In addition to collation of biological evidence, the impact of the CPI synthesis site was evaluated by comparing estimated parameter values in modified CPI models when assuming different fractions of CPI synthesis in the liver (*FL_syn,_* ranged from 0 to 1; i.e., 0–100% of *k_syn_* in the liver).

(12) $$\frac{dC_{\mathrm{blood}}}{\mathrm{dt}}=\left(\left(1‐FL_{\mathrm{syn}}\right)\cdot k_{\mathrm{syn}}‐CL_{R}\cdot C_{\mathrm{blood}}+Q_{H}\cdot \left(C_{\mathrm{LV}}‐C_{\mathrm{blood}}\right)\right)\cdot \frac{1}{V_{c}}$$

(13) $$\frac{dC_{\mathrm{LT}}}{\mathrm{dt}}=\left(\begin{array}{c} FL_{\mathrm{syn}}\cdot k_{\mathrm{syn}}+CL_{passive,u}\cdot \left(\left(C_{\mathrm{LV}}\cdot fu_{b}\right)‐\left(C_{\mathrm{LT}}\cdot fu_{\mathrm{LT}}\right)\right)+CL_{active,u}\cdot C_{\mathrm{LV}}\cdot fu_{b}\\ ‐CL_{B}\cdot C_{\mathrm{LT}}\cdot fu_{\mathrm{LT}} \end{array}\right)\cdot \frac{1}{V_{\mathrm{LT}}}$$

### Theoretical simulation of CPI‐drug interaction with different *CL_active_* and *k_syn_*

CPI PK parameters (baseline, AUCR, maximum concentration (C_max_) observed, and time of maximum concentration observed (T_max_)) were simulated with hypothetical OATP1B inhibitors using the developed CPI population PBPK model. Inhibitors were assumed to have the same PK as rifampicin, but varying OATP1B *Ki* (up to 10‐fold lower potency in inhibition). *CL_active_* and *k_syn_* in the model ranged from 10–125% or 50–125% relative to those in male Caucasians with 521TT, respectively, to capture the covariates estimated in this study. AUCR was calculated as a ratio of AUC_0–24_ with and without inhibitor. To evaluate changes derived from altered CPI disposition, PK of the inhibitor was assumed to be independent of the covariates.

### Statistical analysis

CPI concentration in plasma over whole study period on occasions without rifampicin treatment (C_ave,base_) was calculated for each subject, and mean and SD of C_ave,base_ were calculated for each subpopulation. All occasions without rifampicin were treated as baseline conditions because co‐administration of clinical probes (furosemide) had no effect on plasma CPI of 521TT subjects in study 1 (**Figure** **S1**).

## RESULTS

### Clinical studies evaluating CPI in different OATP1B1 genotype and ethnicity

Men with 521TC (1.86 nM) showed higher C_ave,base_ than 521TT (1.07 ± 0.26 nM) in study 1; the same tendency was also observed in men (TT 0.66 ± 0.11 nM (*n* = 4), TC 1.08 ± 0.14 nM (*n* = 3), and CC 1.74 nM (*n* = 1)) and women (TT 0.54 ± 0.05 nM (*n* = 4), TC 0.69 ± 0.13 nM (*n* = 3), and CC 1.37 nM (*n* = 1)) in study 2. Female subjects showed lower C_ave,base_ than men regardless of OATP1B1 genotype. C_ave,base_ of male Caucasian subjects with 521TT (0.66 ± 0.11 nM in study 2) was lower than those in Asian‐Indians with 521TT genotype (1.07 ± 0.26 nM in study 1 and 0.87 ± 0.16 nM in study 3). C_ave,base_ in male Asian‐Indians with 521TT was comparable between study 1 and study 3.

### Development of CPI population PBPK model with multiple covariates

**Figure 2:**
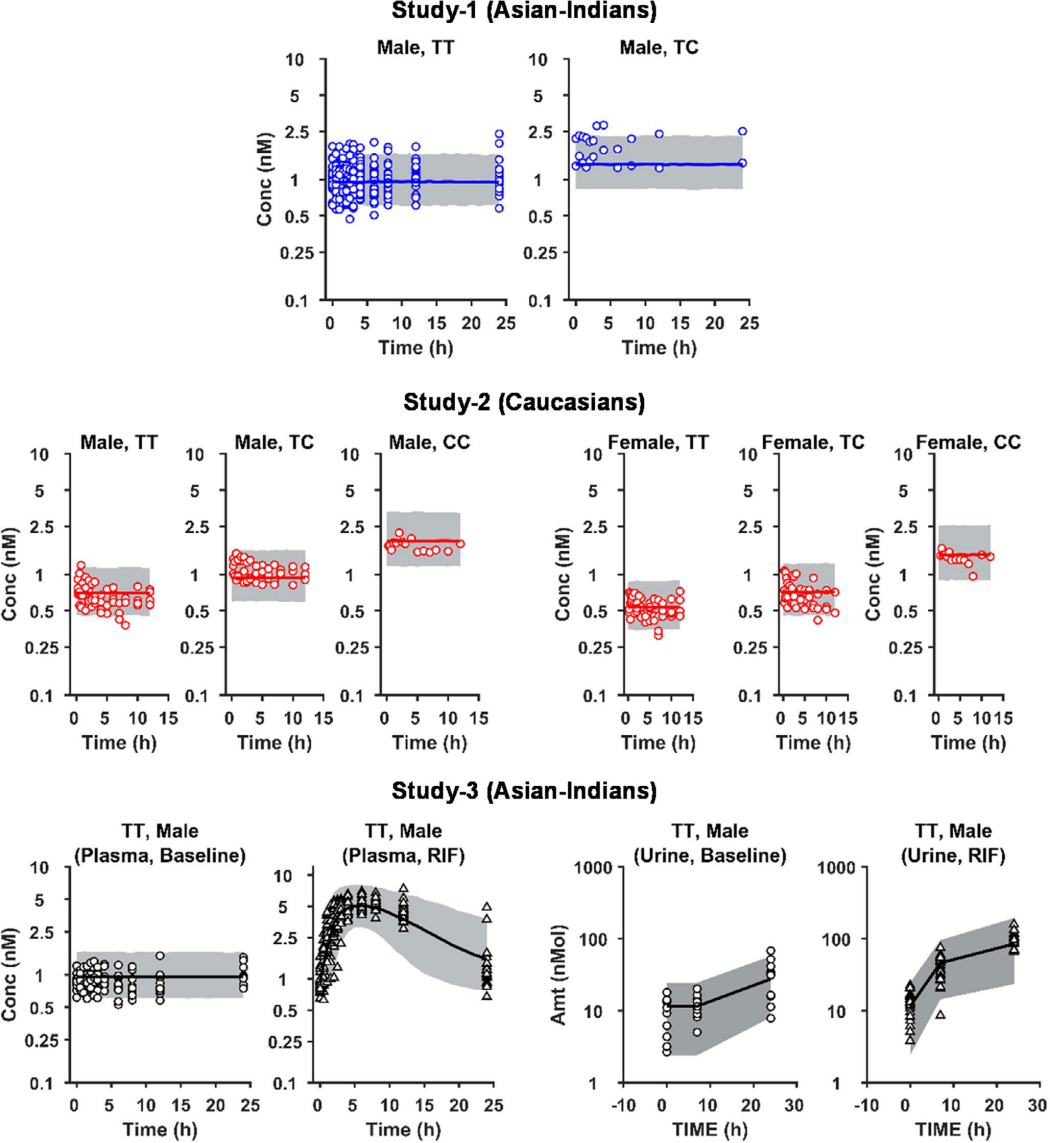
Visual predictive check (VPC) for mechanistic population PBPK modeling of CPI plasma and urine data. Symbols, solid lines, and grey areas represent observed data, median population prediction, and 95% prediction intervals (*n* = 5,000), respectively. Simulations were performed for each subgroup, including subjects with different sex or *SLCO1B1* c.521 (OATP1B1 transporter) genotype (521TT (TT), 521TC (TC), and 521CC (CC)) in three clinical studies. The CPI model with *k_syn_* in the blood (central) compartment was used for the simulation. CPI, coproporphyrin I; PK, pharmacokinetic.

**Figure 3:**
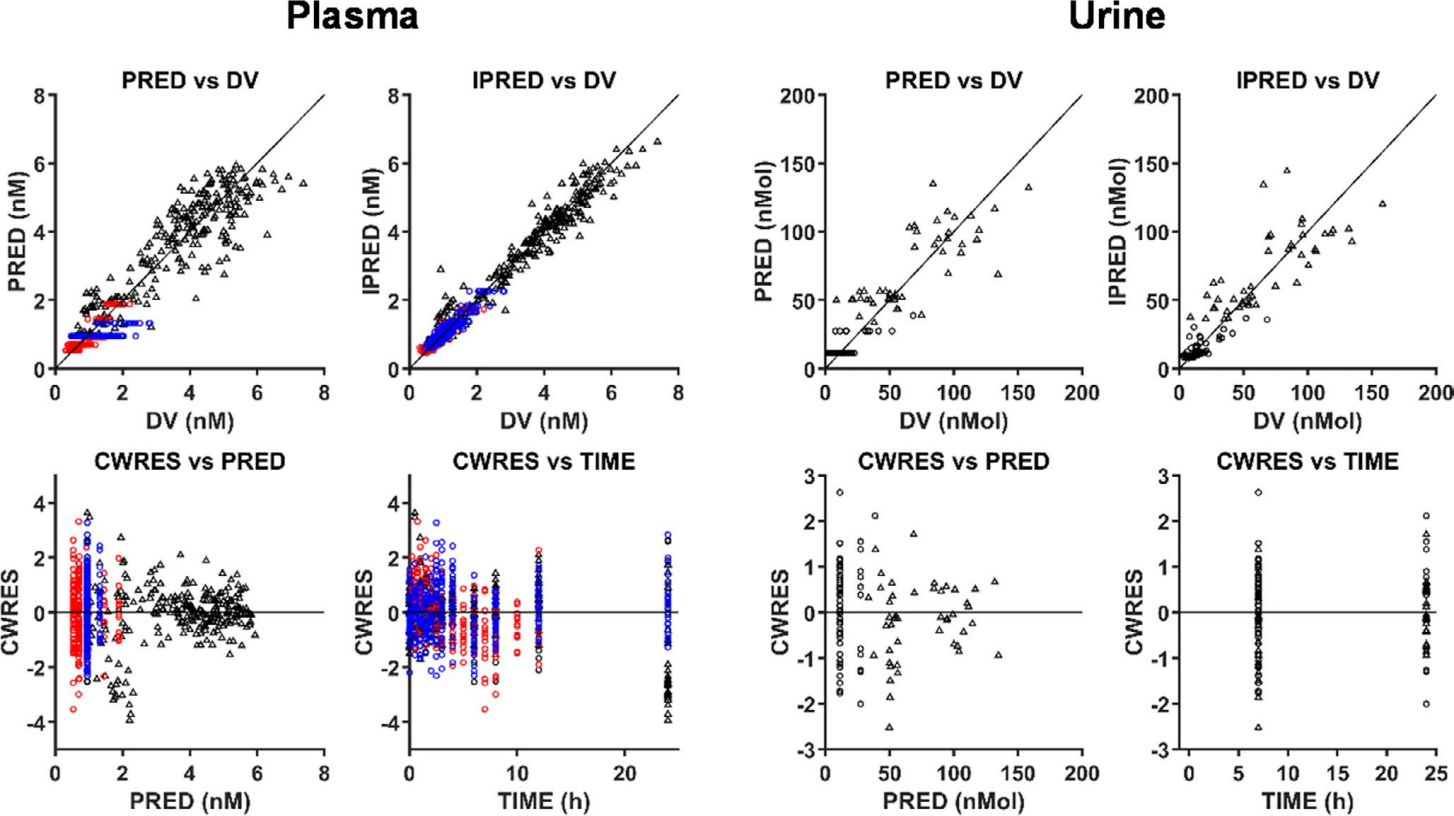
Goodness‐of‐fit (GOF) plots for mechanistic population PBPK modeling of CPI plasma and urine data. Colors represent clinical studies: blue = study 1; red = study 2; and black = study 3. Circles and triangles represent occasions with or without rifampicin administration, respectively. Solid lines are reference lines. The CPI model with *k_syn_* in the blood (central) compartment was used for the simulation. CPI, coproporphyrin I; CWRES, conditional weighted residuals; DV, observed data; IPRED, individual prediction; PRED, population prediction.

The value of *CL_passive,u_* and *f_u,LT_* were fixed to those measured *in vitro* in human hepatocytes (scaled *CL_passive,u_* = 8 L/h or equivalent to 0.76 µL/min/10^6^ cells, and *f_u,LT_* = 0.19, details of *in vitro* studies in **Supplementary Material Section** **S2**). Experimentally determined CPI *f_u,p_* was 0.069. With these parameters fixed, population PBPK CPI model, accounting for the mechanistic liver description and the three covariates, successfully estimated all parameters with acceptable uncertainty (< 35% relative standard error except for additive residual error for CPI in urine; **Table** **2**). The VPC showed adequate coverage of the data, with most of the data within acceptable range in GOF plots, despite moderately smaller conditionally weighted residual for higher PRED (plasma and urine; **Figures** **2** and **3**).

**Table 2:** CPI population PBPK model parameter estimates

Parameter	*k_SYN_* in blood (*FL_syn_* = 0)			*k_SYN_* in liver (*FL_syn_* = 1)		
	Fixed^a^	BSV^b^	BOV^c^	Fixed^a^	BSV^b^	BOV^c^
System parameter						
*k_syn_*, nMol/h	18.4 (11)	10 (30)	‐	33.1 (12)	7.3 (87)	‐
*CL_B_*, L/h	6.24 (24)	38.2 (23)	34.4 (21)	5.28 (8)	16 (28)	14.2 (16)
*CL_R_*, L/h	2.7 (6)	12.7 (30)	‐	2.7 (6)	13.5 (28)	‐
*V_C_*, L	11.9 (21)	25.8 (29)	‐	13 (16)	24.9 (29)	‐
*CL_active,0_*, L/h	1397 (32)	‐	‐	930 (15)	‐	‐
*Ki*, μM	0.93 (7)	‐	‐	1.44 (10)	‐	‐
Covariates						
*FRAX*	0.269 (13)	‐	‐	0.486 (13)	‐	‐
*COV_GEN_* ^d^	0.788	‐	‐	0.673	‐	‐
*COV_RACE_*	0.417 (18)	‐	‐	0.323 (16)	‐	‐
*COV_SEX_*	0.232 (24)	‐	‐	0.26 (21)	‐	‐
Residual unexplained variabilities						
σ_prop_ (%) – plasma	13.2 (5)	‐	‐	13.3 (5)	‐	‐
σ_add_, nM – plasma	0.001 FIXED	‐	‐	0.001 FIXED	‐	‐
σ_prop_ (%) – urine	34.8 (8)	‐	‐	34.9 (8)	‐	‐
σ_add_, nMol – urine	2.3 (50)	‐	‐	2.18 (59)	‐	‐

σ_prop_, proportional residual error; σ_add_, additive residual error; BOV, between occasion variability; BSV, between subject variability; *CL_B_*, biliary clearance; *CL_R_*, renal clearance; *CL_uptake,0_*, hepatic active uptake clearance (*CL_active,u_*) in white men with *SLCO1B1* 521TT genotype; COV*_GEN_*, fractional change in *CL_active,u_* in *SLCO1B1* 521CC genotype; COV*_RACE_*, fractional change in *CL_active,u_* in Asian‐Indian subjectss; *COV_sex_*, fractional change in *k_syn_* in women relative to men; CPI, coproporphyrin I; *FL_syn_*, fraction CPI synthesis in the liver; *FRAX*, surrogate variable of genetic effect; *K_i_*, total rifampicin OATP1B1 inhibition constant (equivalent to 0.10 µM as unbound *K*_i_ calculated with rifampicin *f_u,p_* of 0.11); *k_syn_*, rate of coproporphyrin I synthesis; PBPK, physiologically‐based pharmacokinetic; *V_c_*, volume of blood (central) compartment.

^a^The population (fixed effect) parameters. Values within parentheses represent relative standard errors (RSE, %). ^b^Estimated BSV (%) and its RSE (%). ^c^Estimated BOV (%) and its RSE (%). ^d^Calculated based on the population (fixed effect) parameter estimate of FRAX.

Active process was a major contributor (> 99%) to CPI hepatic uptake; estimated *CL_active,0_* (for the reference 521 TT group) was 1,397 L/h (equivalent to 137 µL/min/10^6^ cells), which was 175‐fold or 517‐fold higher than *CL_passive,u_* or *CL_R_* (2.7 L/h), respectively. Estimated *CL_active,0_* was 2‐fold higher than the CPI *CL_active_* obtained *in vitro* (64.8 µL/min/10^6^ cells, **Table** **S2**). *CL_B_* (6.24 L/h) was lower than previously estimated (12.3 L/h)^18^; however, this finding is not surprising considering the more mechanistic description of CPI hepatic disposition in this analysis, with the inclusion of *CL_active,u_*. The estimated total rifampicin OATP1B *Ki* (0.93 µM, equivalent to 0.10 µM as unbound *Ki* calculated with rifampicin *f_u,p_* of 0.11^24^) was consistent with our previous analysis (1.15 µM).^18^ BSV of *k_syn_*, *CL_B_*, *CL_R_*, and *CL_active,u_* were < 40%.

The parameter estimation revealed 79% lower *CL_active,u_* in 521CC relative to 521TT, which was slightly lower than *in vitro*‐estimated genotype effect based on comparison of CPI uptake in donors with 521TT and 521CC genotype (85.6 ± 6%; **Table** **S3**). Estimated *CL_active,u_* in Asian‐Indians was 42% lower than that in Caucasians. Estimated CPI *k_syn_* in female subjects was 23% lower than that in male subjects.

### Location of CPI synthesis

Our comprehensive literature search on the location of CPI synthesis was not conclusive because of supportive information for both assumptions (details in **Supplementary Material Section** **S4**). In brief, CPI synthesis in blood was qualitatively suggested based on the parallel change in urinary CPI excretion and erythropoiesis activity in the blood.^33^, ^34^ Partial contribution of hepatic CPI synthesis seemed possible because an administered radiolabeled precursor of CPI, which is selectively taken into the hepatic heme synthesis pathway, was partially converted to CPI in humans.^39^

Considering uncertainties above, parameter estimations with different values assumed for *FL_syn_* (from 0 to 1) were investigated. VPC with each set of estimated parameters were visually indistinguishable (estimated values and VPC with *FL_syn_* = 0 (synthesis in blood) and *FL_syn_* = 1 (synthesis in liver) are shown in **Table** **2** and **Figure** **S7** and **S8**, respectively). The model with CPI synthesis in the liver showed comparable parameter estimates to the model assuming CPI synthesis in the blood (central); moderate relative changes in *k_syn_* (+80%), *CL_active,0_* (−33%), *Ki* (+55%), BSV in *k_syn_* (−50%) and *CL_B_* (−82%), and BOV in *CL_B_* (−83%) were evident, with marginal changes in other parameters (±30%). Estimated parameters in the models with different contribution of CPI synthesis in liver (*FL_syn_* = 0.25, 0.5, or 0.75) were between those estimated with *FL_syn_* = 0 and 1 assumptions (**Figure** **S9**).

### Theoretical simulation of CPI‐drug interaction in different covariate groups

**Figure 4:**
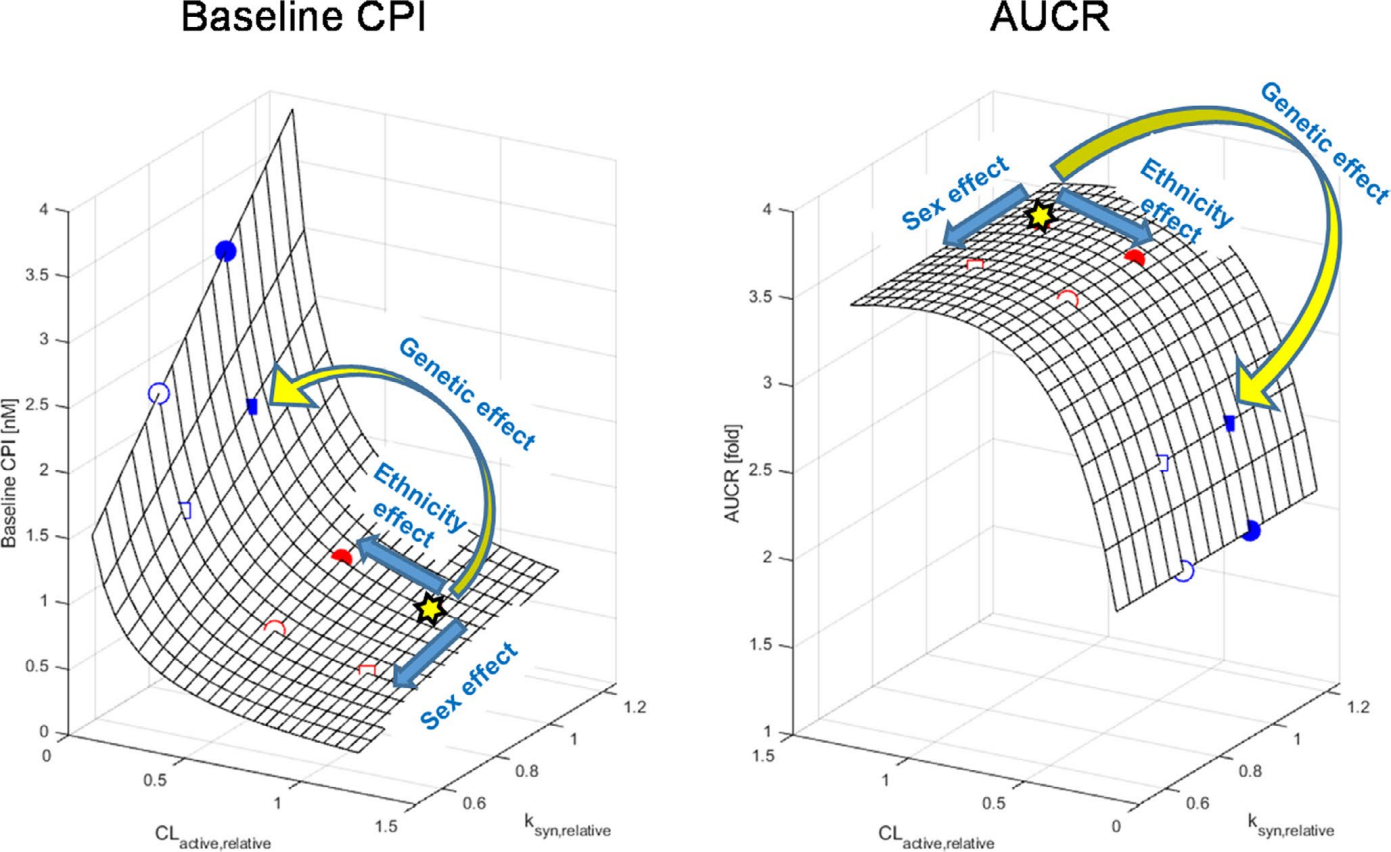
Theoretical simulation of CPI‐drug interaction across different CPI hepatic uptake clearance and synthesis rate. Simulation of CPI baseline and AUCR caused by the rifampicin equivalent OATP1B1 inhibitor with varying CPI hepatic uptake clearance and endogenous synthesis rate, represented as relative values (*CL_active,u,relative_* and *k_syn,relative_*) to those in male Caucasians with 521TT (yellow star), respectively. Symbols on surface plots represent subpopulations simulated with the population (fixed effect) parameters of each subpopulation; men (filled) and women (open), 521TT (red) and 521CC (blue), Caucasians (square), and Asian‐Indians (circle). Yellow and blue arrows indicate effects of three covariates on male white subjects with 521TT. AUCR, fold change in CPI AUC; CL_active_, hepatic active clearance; CPI, coproporphyrin I; k_syn_, zero‐order synthesis.

CPI baseline and C_max_ increased with decreasing *CL_active,u_* (the effect of 521T>C or ethnicity), whereas opposite trend was seen with decrease in *k_syn_* (the effect of sex; **Figure** **4** **and Figure** **S10**). Simulated AUCR with OATP1B inhibitor equivalent to rifampicin decreased with lower initial transporter activity (*CL_active,u_*), whereas AUCR was insensitive to differences in *k_syn_*. The effect of 521T>C genotype on CPI AUCR (25–40% decrease in 521CC relative to 521TT in white and Asian‐Indian subjects, respectively) was more evident than that of ethnicity (2–22% decrease in Asian‐Indian relative to Caucasian subjects in 521TT and CC, respectively). Moderate‐to‐weak OATP1B inhibitors showed the same trends, although the degree of interaction was smaller (**details in Figure** **S11**).

## DISCUSSION

### Utility of model‐informed drug development with CPI model

Mechanistic modeling has been increasingly used to improve quantitative understanding of the formation and elimination mechanisms of endogenous biomarkers for drug transporters; these efforts are more advanced for CPI/OATP1B in comparison to other endogenous biomarkers/transporters. One of the key applications of modeling approaches is to guide optimal design of OATP1B‐mediated interaction studies.^18^, ^35^, ^36^ To that end, modeling and simulation studies have provided information on the adequate sample size to identify weak‐to‐moderate OATP1B inhibitors^18^ and used estimated *in vivo Ki* based on CPI data to simulate the DDI between rifampicin/new inhibitors and statins.^35^, ^36^, ^40^ One of the key advantages of CPI as an endogenous biomarker of OATP1B function *in vivo* is its relatively stable baseline in contrast to other biomarkers (e.g., fatty or bile acids and their conjugates).^15^ Recent clinical studies have provided evidence of the effect of OATP1B1 genetic polymorphism^25^, ^28^, ^31^ on CPI plasma concentration. Therefore, this study aimed to expand our previous CPI model^18^ to incorporate multiple covariates on CPI disposition and investigate their impact on the evaluation of CPI‐drug interaction risk.

CPI population PBPK model was based on several assumptions. Certain parameters (e.g., *CL_passive,u_* and *f_u,LT_*) were fixed to the values measured *in vitro* to improve parameter identifiability (**Supplementary Material Section** **S2**). Enterohepatic circulation of CPI was not considered in our model, supported by the recent studies.^29^, ^30^ For pragmatic reasons, concentration of rifampicin in the central compartment was used as inhibitory concentration affecting CPI *CL_active,u_*; any potential rifampicin concentration gradient between blood and hepatic inlet was not considered. This assumption may potentially underestimate rifampicin OATP1B *Ki*, but the covariates estimated as relative changes are not likely to be sensitive to this assumption. Inhibition of MRP2 by rifampicin was not considered due to the lack of bile data for verification, as discussed previously.^18^ Biological evidence for the location of CPI synthesis was inconclusive (**Supplementary Material Section** **S4**) and CPI plasma exposure is likely to be derived from both liver and blood. Parameter estimation with assumed CPI synthesis either in the blood or liver resulted in comparable parameters (**Table** **2**) and recovery of plasma data. These findings are not surprising and highlight that plasma data in isolation (or combined with urine) are not sufficiently sensitive/informative to differentiate the CPI synthesis site, in particular, any contribution of the liver.

### Covariates affecting CPI hepatic active uptake clearance

In our study, OATP1B1 521T>C was considered a sole allelic variant that affects CPI disposition, due to limited clinical evidence of the effect of other polymorphisms (e.g., c.388A>G).^28^, ^31^ The implementation of the genetic effect in the model was based on relatively small number of subjects with c.521T>C mutation (9 subjects with 521TC or CC), but the overall trends are in line with a recently published study.^31^ The 521CC has resulted in 79% lower CPI *CL_active,u_* relative to 521TT, in agreement with clinical OATP1B1 probes.^11^, ^41^ The 521CC effect was slightly lower compared with *in vitro* evaluation (80–92%; **Table** **S3**). This slight discrepancy can be rationalized by large interindividual differences in protein expression of OATP1B^42^, ^43^ and small number of hepatocyte donors used in the *in vitro* evaluation. Considering available dataset, this genetic effect on CPI exposure was mainly based on the data reported in Caucasians. Ethnic differences in the degree of genetic effect are not likely considering comparable effects of 521T>C mutation reported for clinical OATP1B probes between Caucasians and Asians.^41^

The literature evidence of ethnic differences in the activity of OATP1B relative to Caucasians was reported for Japanese,^11^ but not for Asian‐Indians due to limited number of clinical studies in this population. Two clinical studies reported higher (up to 63%) AUC of rosuvastatin in Asian‐Indians relative to Caucasians^7^, ^9^; however, BCRP 421G>A status of those subjects was not reported. Therefore, those results require careful interpretation, as rosuvastatin is a substrate of OATP1B1 and BCRP and genetic polymorphism of both transporters affects its plasma exposure.^7^, ^8^, ^9^, ^44^ Birmingham *et al*.^9^ reported moderately (> 26%) higher rosuvastatin AUC in Asian‐Indians relative to Caucasians regardless of OATP1B1 and BCRP genotype, implying the existence of intrinsic ethnic variability in OATP1B activity between the two populations. Therefore, the ethnicity effect in the CPI model was assumed on *CL_active,u_* and 42% lower *CL_active,u_* was estimated for Asian‐Indians relative to Caucasians. We excluded the possibility of the ethnicity effect on *k_syn_* because of comparable blood hemoglobin levels reported for Asian‐Indians and Caucasins,^45^ suggesting minimal differences in CPI synthesis between these two populations. Reported mean CPI C_ave,base_ in Japanese (men, 521TT) of 0.63–0.77 nM^26^, ^31^ is comparable to Caucasians (**Table** **1**) and in contrast to the proposed ethnic differences in OATP1B activity between Japanese and Caucasians.^11^ Lower hemoglobin has been reported in Japanese relative to Caucasians, which may imply lower CPI synthesis in Japanese.^46^

### Covariates affecting CPI synthesis rate

In Caucasian subjects, lower C_ave,base_ was observed in women relative to men regardless of OATP1B1 genotype (**Table** **1**). Comparable exposure of other endogenous biomarkers of OATP1B (e.g., fatty acids) between men and women in the same subjects (data not shown) suggested no sex effect on *CL_active,u_*, but reduced CPI synthesis in women. These findings are supported by the comparable protein expression of hepatic OATP1B between men and women^42^ and lower blood hemoglobin in women.^47^ Although this study used all the available individual CPI data, it is worth noting that small sample size in some subpopulations in this study (e.g., 521CC) may bias the estimation of covariates, as suggested by power calculations in previous studies.^18^ Further studies with larger sample size, including different demographics (e.g., OATP1B1 genotype, ethnicity, or sex) would address these questions and validate/confirm assumptions made here with regard to CPI synthesis or intrinsic ethnic differences in OATP1B activity.

### Prospective application of the developed CPI model

As CPI is a surrogate marker of OATP1B functional activity *in vivo*, the population PBPK model developed here provides a powerful and informative tool for the prospective evaluation of OATP1B‐mediated interaction risk. This approach is particularly valuable for exploration of interaction risk in subpopulations that are often not considered in clinical studies or in populations with multiple co‐existing covariates. Simulations of such what‐if scenarios are where a model‐informed drug development approach exerts its true potential. Theoretical simulations of CPI‐drug interactions illustrated complex interplay between biomarker synthesis and OATP1B‐mediated uptake, with high sensitivity of AUCR to changes in *CL_active,u_*, but not to *k_syn_* (**Figure** **4**). Decrease in *CL_active,u_* reduces the proportion of CPI eliminated via OATP1B, which is compensated by increased elimination of CPI into urine (data not shown), resulting in decreased AUCR. Relatively greater effect of 521T>C genotype on *CL_active,u_* (−79% in 521CC relative to 521TT) than that of ethnicity (−42% in Asian‐Indian relative to white subjects) results in more pronounced impact of the genetic effect on the CPI interaction (AUCR). Simulated decreased AUCR in 521CC is consistent with data reported with clinical OATP1B probes^3^, ^6^ and suggests the risk of underestimation of the magnitude of transporter‐mediated interaction if these subjects are included in a CPI‐drug interaction study, which is more likely to happen in clinical studies performed without prior genotyping (e.g., first‐in‐human study) or in populations with higher prevalence of 521CC.^48^ In contrast, any changes in CPI synthesis did not affect fraction transported via OATP1B and simulated AUCR, highlighting no risk of underestimation of the OATP1B interaction magnitude if female subjects are included in the study. As exemplified here, the modeling of CPI gives us prospective insights of clinical output and enables optimal clinical design to increase likelihood of success. Furthermore, the physiological description of the liver processes in the CPI model enables the extension of the model to other special populations (e.g., evaluation of the changes in OATP1B activity in the chronic kidney disease population).^49^, ^50^

In conclusion, this study evaluated the impact of the genotype of OATP1B1 c521T>C, ethnicity, and sex on CPI baseline and its OATP1B‐mediated interaction potential. For the first time, this revised CPI population PBPK model implemented reduced active uptake due to OATP1B1 polymorphism, ethnic differences in active uptake between Asian‐Indians and Caucasians, and reduced CPI synthesis in women. Modeling highlighted that assumption of the CPI synthesis site (blood, liver, or both) has marginal impact on the simulated plasma exposure of this endogenous biomarker ; availability of liver concentrations would be necessary to allow differentiation of these sites. The CPI PBPK model proposed here aims to facilitate the design of prospective clinical studies with OATP1B perpetrator drugs to maximize the sensitivity of this biomarker.

## Funding

H.T. was financially supported by a fellowship grant from Asahi Kasei Pharma Corporation. S.B. was supported by a PhD studentship from the Biotechnology and Biological Sciences Research Council, UK (BB/L502376/1) and UCB, UK.

## Conflict of interest

The authors declared no competing interests for this work.

## Author contributions

H.T., S.B., K.M., H.S., K.O., and A.G. wrote the manuscript. H.T., S.B., H.S., K.O., and A.G. designed the research. H.T., S.B. and Y.Z. performed the research. H.T., S.B., Y.Z., and K.O. analyzed the data.

## Supporting information

Supplementary Material

Supplementary Material
