# Supplementary material for: PBPK Model of Coproporphyrin I: Evaluation of the Impact of SLCO1B1 Genotype, Ethnicity, and Sex on its Inter‐Individual Variability
Source: CPT Pharmacometrics Syst Pharmacol. 2021 Jan 19;10(2):137–47. doi: 10.1002/psp4.12582 (PMC7894406; doi:10.1002/psp4.12582)
Supplement: Supplementary file 1 — Supplementary Material [file PSP4-10-137-s001.docx]

**Supplementary Material for “Application of PBPK model for coproporphyrin I to evaluate the impact of SLCO1B1 genotype, ethnicity, and sex on its inter-individual variability”**

Hiroyuki Takita^1,2^, Shelby Barnett^1^, Yueping Zhang^3^, Karelle Ménochet^4^, Hong Shen^3^, Kayode Ogungbenro^1^ and Aleksandra Galetin^1*^

^1^Centre for Applied Pharmacokinetic Research, Division of Pharmacy and Optometry, School of Health Sciences, Faculty of Biology, Medicine and Health, University of Manchester, Manchester, M13 9PT, UK

^2^Laboratory for Safety Assessment and ADME, Pharmaceuticals Research Center, Asahi Kasei Pharma Corporation, Shizuoka, Japan

^3^Pharmaceutical Candidate Optimization, Bristol-Myers Squibb, Princeton, New Jersey

^4^Quantitative Pharmacology & DMPK, UCB, Slough, UK

Contents

[1. Details of Study-1 2](#_Toc47730148)

[2. *In vitro* studies for Coproporphyrin I 4](#_Toc47730149)

[3. POPULATION PBPK model for Coproporphyrin I 12](#_Toc47730150)

[4. Location of coproporhyrin I synthesis 18](#_Toc47730151)

[5. Theoretical simulation of CPI-drug interaction in sub-populations 23](#_Toc47730152)

[References 25](#_Toc47730153)

# **Details of Study-1**

Study-1 was previously reported as a clinical study to evaluate the drug-drug interaction between probenecid and furosemide^1^. In brief, the study was an open-label, three-period, crossover study in 14 healthy male Asian-Indian subjects. Subjects received 1000-mg probenecid tablets (Bencid; Geno Pharmaceuticals Limited), 40-mg furosemide tablets (Lasix; Sanofi India Limited), and 1000-mg probenecid tablets plus 40-mg furosemide tablets in periods 1, 2, and 3, respectively. CPI concentrations in plasma samples collected prior period1 (predose) and post furosemide treatment were measured by LC-MS/MS, as described in Lai *et al*.^2^. Samples were protected from light until thought the study. The studies were conducted in accordance with all applicable regulatory and Good Clinical Practice guidelines and followed the ethical principles originating in the Declaration of Helsinki.

Genotype of *SLCO1B1* c.521 T>C was evaluated by polymerase chain reaction (PCR). The probe sets were obtained from Thermo Fisher Scientific (Grand Island, NY). Taqman assay was performed using 5 µL of iQ multiplex powermix (Bio-Rad, Hercules, CA), 0.5 µL of probe mix, 3.5 µL of nuclease-free water, and 1 µL of DNA eluate from each sample. PCRs were conducted in triplicate in a 384-well hard-shell plate using the CFX 384 real time PCR detection system from Bio-Rad. Reaction conditions were as follows: enzyme activation at 95°C for 3 minutes, denaturation at 95°C for 15 seconds, and annealing/extension at 60°C for 1 minute and for a total of 50 cycles. The probes contained a fluorescent reporter dye (VIC specific for allele 1, and FAM specific for allele 2) attached to its 5′ end and a quencher dye at its 3′ end. Allelic discrimination was analysed based on relative fluorescence from the probe sets using CFX manager software from Bio-Rad. The presence of a VIC-only fluorescent signal represents homozygosity for allele 1 (521T), and an FAM-only fluorescent signal indicates homozygosity for allele 2 (521C). The presence of both signals suggests heterozygosity of alleles 1 and 2.


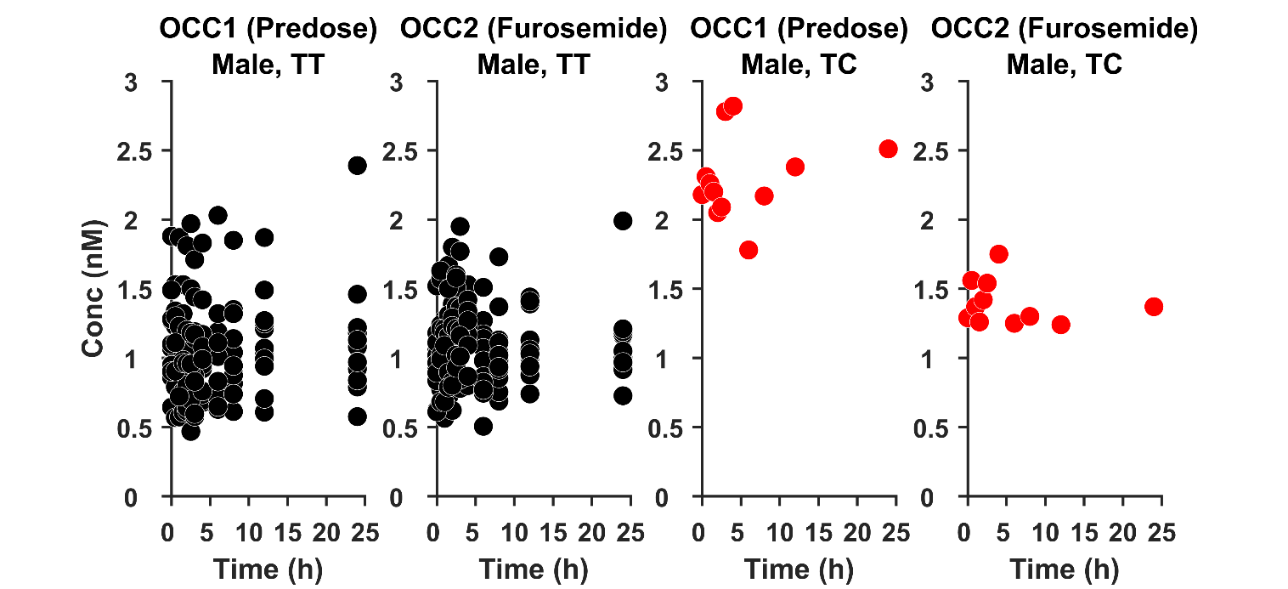


**Figure S1 Plasma concentrations of coproporphyrin I in Study-1**

Scatter plots of plasma concentration of coproporphyrin I in two occasions (OCC) of Study-1: OCC1; predose, OCC2; furosemide. The numbers of subjects and plasma samples are in **Table 1**.

# ***In vitro* studies for Coproporphyrin I**

## **Evaluation of uptake kinetics of CPI using human hepatocytes**

### **Materials**

Dulbecco's phosphate-buffered saline (DPBS) (Catalogue #14040-224) and penicillin-streptomycin were purchased from ThermoFisher (Loughborough, UK). Bovine serum albumin (BSA), rifampicin, naloxone, dimethyl sulphoxide (DMSO) and insulin were purchased from Sigma Aldrich (Dorset, UK). High performance liquid chromatography (HPLC) grade methanol and collagen type I coated 24‑well plates (Catalogue #734-0277) were purchased from VWR International (Lutterworth, UK). Paritaprevir was purchased from Medchem Express (New Jersey, USA). Coproporphyrin I dihydrochloride was purchased from Frontier Scientific (Carnforth, Lancashire UK). Human hepatocytes (four donors: QQE, DSX, IPH, and NHI), *InVitroGRO*™ CP plating medium (Catalogue #Z990003), and Torpedo™ Antibiotic Mix were purchased from BioIVT (Neuss, Germany). Donor demographics are highlighted in **Supplementary Table S1**.

**Table S1** **Human hepatocyte donor demographics**

| Donor | Sex | Age | Ethnicity | BMI | Smoker | Alcohol Use | Drug Use | Medication | Cause of Death | *SLCO1B1* c.521 Genotype |
| --- | --- | --- | --- | --- | --- | --- | --- | --- | --- | --- |
| QQE | F | 42 | Caucasian | 32.2 | No | Yes | No | Adderall | Anoxia | TT |
| DSX | F | 56 | Hispanic | 25.9 | Yes –quit 10 years ago | No | No | None | Cerebrovascular accident | TT |
| IPH | F | 52 | Caucasian | 28.8 |  | No | No | No | Anoxia | TT |
| NHI | M | 48 | Caucasian | 23.3 | Yes | Yes | No | Sybostatin | Anoxia | CC |

### **Culture of hepatocyte**

Human hepatocytes were prepared as per supplier’s instructions, using a medium containing *InVitroGRO*™ CP plating medium and *Torpedo*™ Antibiotic Mix (45:1). Hepatocyte viability was determined using the trypan blue exclusion method, with a viability threshold of >85%. Cells were then re‑suspended to a seeding density of 700,000 cells/mL, seeded on collagen type I coated 24-well plates at 350,000 cells/well. Cells were cultured for 4 hours at 37°C, with 5% CO_2_ to allow sufficient attachment for a confluent monolayer.

### **Uptake of CPI in plated human hepatocytes**

The human hepatocyte donor QQE (*SLCO1B1* c.521 TT, wild type) was used to assess the uptake kinetics of CPI over multiple concentrations and time points. Briefly, following attachment to collagen coated plates wells, hepatocytes were washed twice with 400 µL of DPBS (37°C), then pre-incubated with 1 mM 1-Aminobenzotriazole for 20 minutes. Following incubation with ABT, buffer was removed and a range of CPI concentration were added (0.1‑30 µM). After the uptake period (0.5, 2, 5, 20, 60, and 150 minutes), a washing protocol was applied, described as follows. To stop uptake wells were quenched with 800 µL 0.2% BSA in DPBS (ice cold), washed twice with 800 µL 0.2% BSA in DPBS (37 °C) and then once with 800 µL DPBS (37 °C). Finally cells were lysed with 200 µL H_2_O and frozen (‑20 °C) over night. Protein measurement in lysate samples were conducted using a BCA assay. Considering the instability of CPI in light^3^, experiments were conducted under dim light conditions and once prepared; samples were protected from light until and during LC-MS/MS analysis. LC-MS/MS analysis of CPI was implemented as reported previously^4^.

### **Estimation of CPI *in vitro* kinetic uptake parameters**

To estimate the kinetic uptake parameters for CPI, a two‑compartment mechanistic model was used, as described in^5^. Briefly, this approach involved simultaneous fitting of cellular and medium concentrations to estimate the maximum uptake rate (*V_max_*), affinity constant (*K_m,u_*), bidirectional passive diffusion clearance (*CL_passive,u_*) and the unbound fraction in the cell (*fu_cell_*). Active uptake clearance (*CL_active,u_*) was then calculated from the ratio of *V_max_* over *K_m,u_*. Fitting of cellular uptake data (Eq S1 and S2) was done in MATLAB R2016a (MathWorks, MA), where the unbound medium concentration is denoted by *C_med,u_* and the volume of the medium (*V_med_*) is the incubation volume of 400 µL. The volume of the cell (*V_cell_*) used for human hepatocytes was 2.7 µL/10^6^ cells^6^. To support the robustness of the *fu_cell_* estimation, kinetic studies were performed over the range of CPI concentrations and time points under cell-medium steady-state conditions, as reported previously^5^.

$\frac{{dC}_{cell}}{dt}=\frac{\frac{V_{max}\cdot C_{med,u}}{K_{m,u}+C_{med,u}}+{CL}_{passive,u}\cdot C_{med,u}-{CL}_{passive,u}\cdot C_{cell}\cdot{fu}_{cell}}{V_{cell}}$ Eq S1

$\frac{\mathrm{dC}_{med,u}}{\mathrm{dt}}=\frac{-\frac{V_{\max}\cdot C_{med,u}}{K_{m,u}+C_{med,u}}-\mathrm{CL}_{passive,u}\cdot C_{med,u}+\mathrm{CL}_{passive,u}\cdot C_{\mathrm{cell}}\cdot\mathrm{fu}_{\mathrm{cell}}}{V_{\mathrm{med}}}$ Eq S2

The *CL_passive,u_* was fixed to 0.76 µL/min/10^6^ cells, which was calculated from the intrinsic clearance of CPI at 30 µM assuming saturation of active uptake and passive clearance being the dominant route. This *CL_passive,u_* was also reproduced in independent *in vitro* studies with multiple potent OATP1B1 inhibitors (rifampicin or cyclosporine, internal data).This approach was required to improve parameter estimates for *fu_cell_*, as *CL_passive,u_* and *fu_cell_* are highly correlated within the model. In addition, standard errors (and relative standard errors – CV%) for parameters were also derived following parameter estimation. Confidence in parameter estimates were categorised as high, moderate and low, with CVs of <30%, <60% and >60 %, respectively.


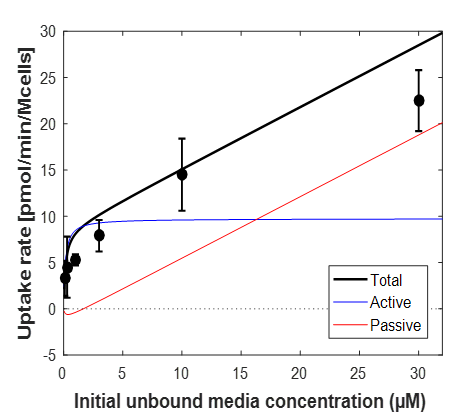


**Figure S2** **Kinetic plot of CPI uptake in plated human hepatocytes**

Kinetic plot of CPI uptake in plated human hepatocytes (QQE) over a concentration range (0.1‑30 µM). Total, active, and passive uptake are represented by the black, blue, and red lines, respectively. The black dots represent the mean uptake rate ± standard deviation of CPI over 5 minutes for a given concentration.

**Figure S3 Goodness-of-fit (GOF) plots of model fitting to CPI cellular uptake data in human hepatocytes (QQE)**

A) Observed and predicted cellular concentration time profiles B) Predicted vs observed cellular concentrations, solid black line represents the line of unity and dashed line represent 2-fold error lines C) Weighted residuals vs time D) Individual weighted residual vs individual prediction.

**Table S2** ***In vitro* kinetic uptake estimates for CPI**

| **Parameter** | **Estimate** |
| --- | --- |
| *V_max_* (pmol/min/10^6^ cells) | 9.83 (14%) |
| *K_m,u_* (µM) | 0.15 (26%) |
| *CL_passive,u_* (µL/min/10_­_­^6^ cells) | 0.76 |
| *fu_cell_* | 0.19 (12%) |
| *CL_active,u_* (µL/min/10^6^ cells) | 64.77 |
| % Active | 98.8 |

Parameters obtained from mechanistic modelling of cellular uptake data in plated human hepatocytes (QQE). The numbers in parenthesis refer to the coefficient of variation (CV) estimated for each parameter.

### **Uptake of CPI in plated hepatocytes with different OATP1B1 genotypes**

In addition to the extended uptake data generated in donor QQE (521TT), uptake of CPI was assessed at a single concentration in donors DSX and IPH (521TT), and NHI (521CC) in order to assess the differences in active uptake between *SLCO1B1* 521TT and CC genotypes. Uptake was assessed at a single CPI concentration of 0.3 µM and over 5 minutes; these conditions were selected to ensure CPI detection in the lysate by LC‑MS/MS. Addition of 400 µL of CPI initiated uptake, which was terminated at 0.5, 1, 1.5, 2, and 5 minutes using the washing protocol described above.

Total uptake clearance (*CL_uptake,T_*) for CPI at 0.3 µM in donors DSX, IPH, and NHI was calculated using the concentration measured in the lysate over 5 minutes (Eq S3). An analogous approach was performed using uptake data generated in a wild type *SLCO1B1* donor (QQE). Only uptake data generated at 0.3 µM over 5 minutes was used from QQE to allow direct comparison to single concentration donors (DSX, IPH, and NHI).

${CL}_{uptake,T}=\frac{\left( \frac{S\times V}{P} \right)}{C}$ Eq S3

Where *S* represents the uptake slope in the lysate (nM/min), *V* is the lysate volume of the lysate (mL), *P* is the protein concentration/number of cells (mg/mL or 10^6^ cells) and *C* is the concentration of CPI (µM). In order to calculate the *CL_active,u_* in each donor, the *CL_passive,u_* was subtracted for the total uptake value. The *CL_passive,u_* value estimated for donor QQE (0.76 µL/min/10^6^ cells) was used, assuming that this parameter is constant between donors. Following the calculation of *CL_active,u_* the percent reduction in *CL_active,u_* in the CC donor (NHI) was calculated. Significant differences in *in vitro* CPI uptake between hepatocytes with 521TT and CC was evaluated by student’s t-test (p<0.05).

**Figure S4 Uptake of CPI in hepatocytes with different genotype of *SLCO1B1***

Uptake of CPI at 0.3 µM in plated human hepatocytes using *SLCO1B1* 521TT (QQE ●, DSX ♦, IPH ●) and 521CC (NHI ◼). Each time point was performed in triplicate and is presented as mean ± standard deviation.

**Table S3 Comparison of CPI active uptake and passive clearance in plated human hepatocytes at a single concentration (0.3 µM), using SLCO1B1 521TT and 521CC donors**

| Parameter  (genotype of SLCO1B1) | QQE  (521TT) | DSX  (521TT) | IPH  (521TT) | NHI  (521CC) |
| --- | --- | --- | --- | --- |
| *CL_uptake,T_* (µL/min/10^6^ cells) | 14.89 | 6.32 | 8.62 | 1.90* |
| *CL_passive,u_*^+^  (µL/min/10^6^ cells) | 0.76 | 0.76 | 0.76 | 0.76 |
| *CL_active_* (µL/min/10^6^ cells) | 14.13 | 5.56 | 7.86 | 1.14 |
| Decrease in *CL_active_* in 521CC relative to 521TT (%) | 92 | 80 | 85 | - |
| * p<0.05, ^+^ CL_passive,u_ value obtained in single donor QQE from uptake studies in human hepatocytes. | | | | |

## **Plasma protein binding**

CPI plasma protein binding was determined by equilibrium dialysis method in 96-well dialysis plate (HTDialysis, Gales Ferry, CT) with 12-14 kDa molecular weight cut-off cellulose membranes. In brief, 150 µL of undiluted 4xcharcoal stripped human plasma spiked with ^15^N_4_-CPI (Toronto, Research Chemicals, Toronto, ON, Canada) was added into one side of compartment in a chamber in triplicate at final concentration of 1 nM, whereas 150 µL of phosphate buffer (0.1 M, pH 7.4) was added into the other compartment of the dialysis cells in the same manner. The cells were then sealed, attached to a rocking shaker and rotated (2,500 rpm) in a 37⁰C incubator with 10% CO_2_ for 5 hours. After dialysis, the contents of the two compartments of the dialysis cells were collected separately. An opposite matrix was added into plasma or buffer side samples to make a uniformed matrix. Calibration standard was prepared in the same matrix. Then equal volume of internal standard (^15^N_4_-CPI) working solution contain 6 M formic acid was added to the samples, vortex and load on a 96-well Biotage Isolute SLE+ plate. Following this, three 500 µL of ethyl acetate were added to each well to elute. After final elution, samples were evaporate under nitrogen stream and reconstituted with 60 µL of water / acetonitrile (80%: 20%, v/v) containing 1 M formic acid.

Concentrations of CPI in plasma and buffer were determined by LC-MS/MS using the method developed previously^7^. In brief, a Triple Quad™ 6500 mass spectrometer with a TurboIonSpray™ (TIS) source from Sciex (CA, USA) equipped with an UPLC system consisting of two Shimadzu binary pumps LC-30AD and SIL-30ACMP auto sampler (Kyoto, Japan). The chromatographic separation was performed on an Acquity™ UPLC BEH C18 (2.1 × 100 mm, 1.8 µm) column and a VanGuard BEH C18 (2.1 × 10 mm, 1.7 μm) guard column, both purchased from Waters (MA, USA). The mobile phases A and B consisted of 0.1% formic acid in water and 0.1% formic acid in acetonitrile. The system was held at 60°C with a constant flow of 0.5 mL/min using a short gradient. Quantitation was achieved by MS/MS detection in positive multiple reaction monitoring (MRM) mode for both CPI and ^15^N_4_-CPI. The MRM precursor/product ion transitions were as follows: m/z 655.4 → 596.3 for CPI and m/z 659.3 → 600.3 for internal standard ^15^N_4_-CPI. The source temperature and ion spray voltage were set at 450°C and 4000 volts, respectively. The other source parameters setting were: curtain gas, 30 psi; CAD gas, 8 psi; gas 1, 75 units; gas 2, 60 units. The chromatographic peak integration and data processing were performed using Analyst® software (version 1.6.2, AB SCIEX)

The percentage of plasma protein bound, stability and recovery are calculated as shown below:

% Protein binding = (1-(C_u_/C_p_)) x 100% Eq S4

where C_u_ is the ^15^N_4_-CPI concentration in buffer and C_p_ is the analyte concentration in plasma at the end of incubation.

% stability = (C_5h_ / C_0h_) x 100% Eq S5

where C_0h_ is initial ^15^N_4_-CPI concentration in plasma, C_5h_ is the concentration at the end of incubation at 37⁰C.

% recovery = ((C_u_+C_p_) / C_0h_) x 100% Eq S6

# **Population PBPK model for Coproporphyrin I**

## **Model parameters**

**Table S4 Fixed physiological and CPI dependent parameters used in the CPI PBPK model**

|  | Parameter | Symbol | Value | Unit | Reference |
| --- | --- | --- | --- | --- | --- |
| System parameter | Hepatic blood flow | *Q_H_* | 92.7 | L/h | Valentin (2002)^8^ |
|  | Volume of liver blood | *V_LB_* | 0.184 | L | Valentin (2002)^8^ |
|  | Volume of liver tissue | *V_LT_* | 1.6 | L | Valentin (2002)^8^ |
| CPI Specific parameter | Fraction unbound in liver tissue | *fu_LT_* | 0.19 | - | fu_cell_ in CPI uptake assay |
|  | Fraction unbound in the plasma | *fu_p_* | 0.069 | - | Evaluated in protein binding assay |
|  | Fraction unbound in the blood | *fu_b_* | 0.11 | - | fu_p_/B:P |
|  | Blood to plasma ratio | *B:P* | 0.628 | - | Yoshikado *et al*. (2018)^9^ |
|  | Hepatic passive clearance | *CL_passive,u_* | 0.76 | µL/min/10^6^ cells | Estimated in CPI uptake assay |

## **Model predictability of individual CPI plasma and urine data**

Individual CPI in plasma


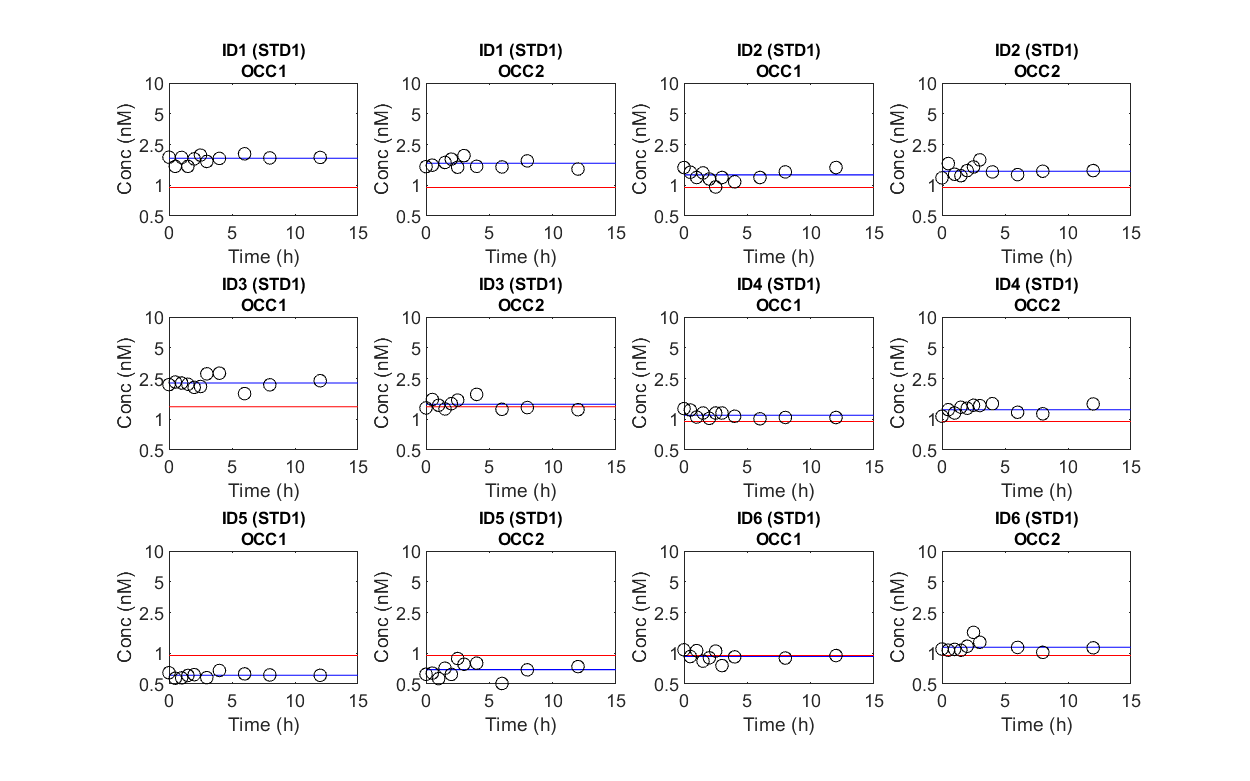

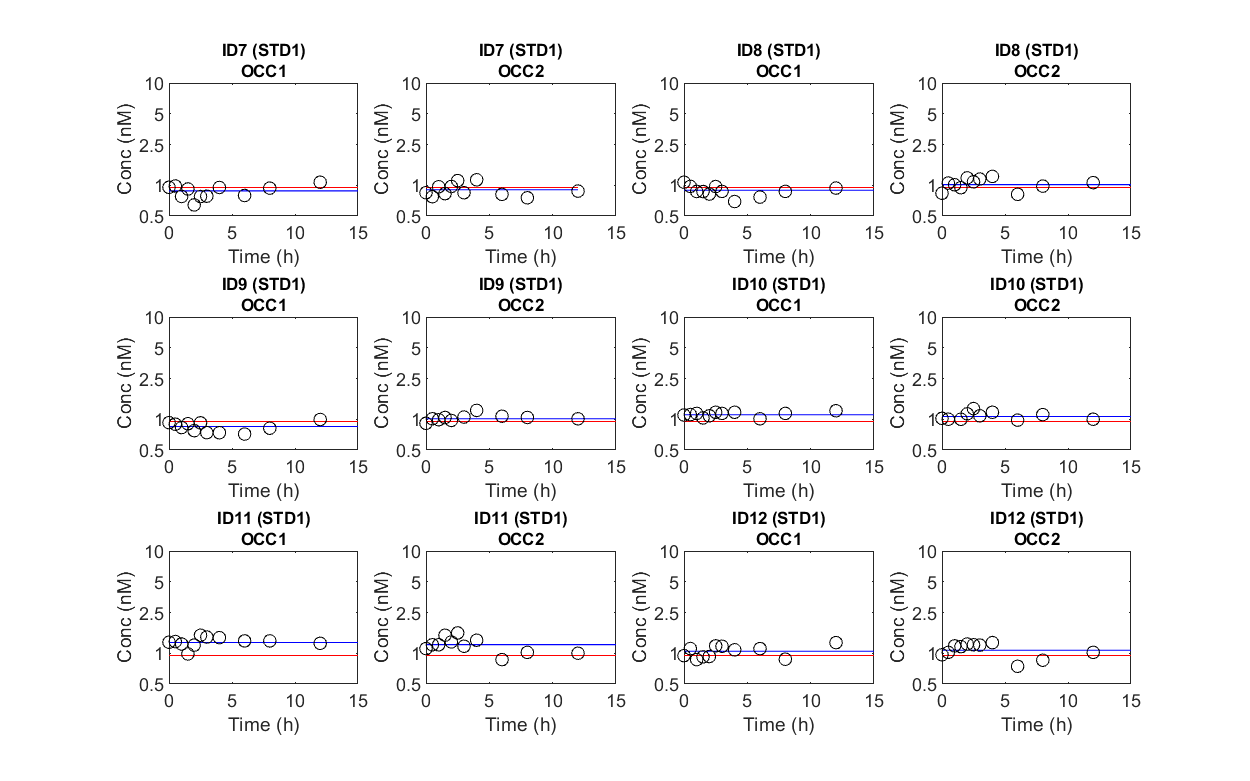

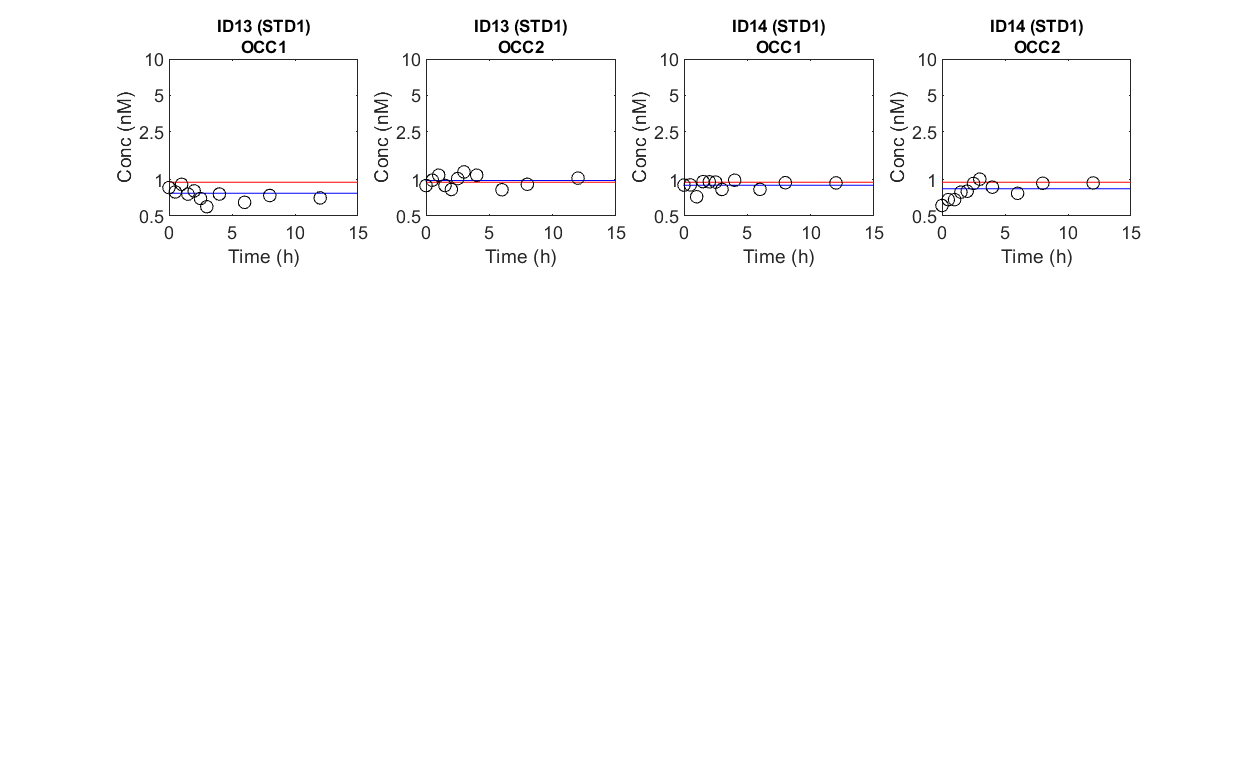

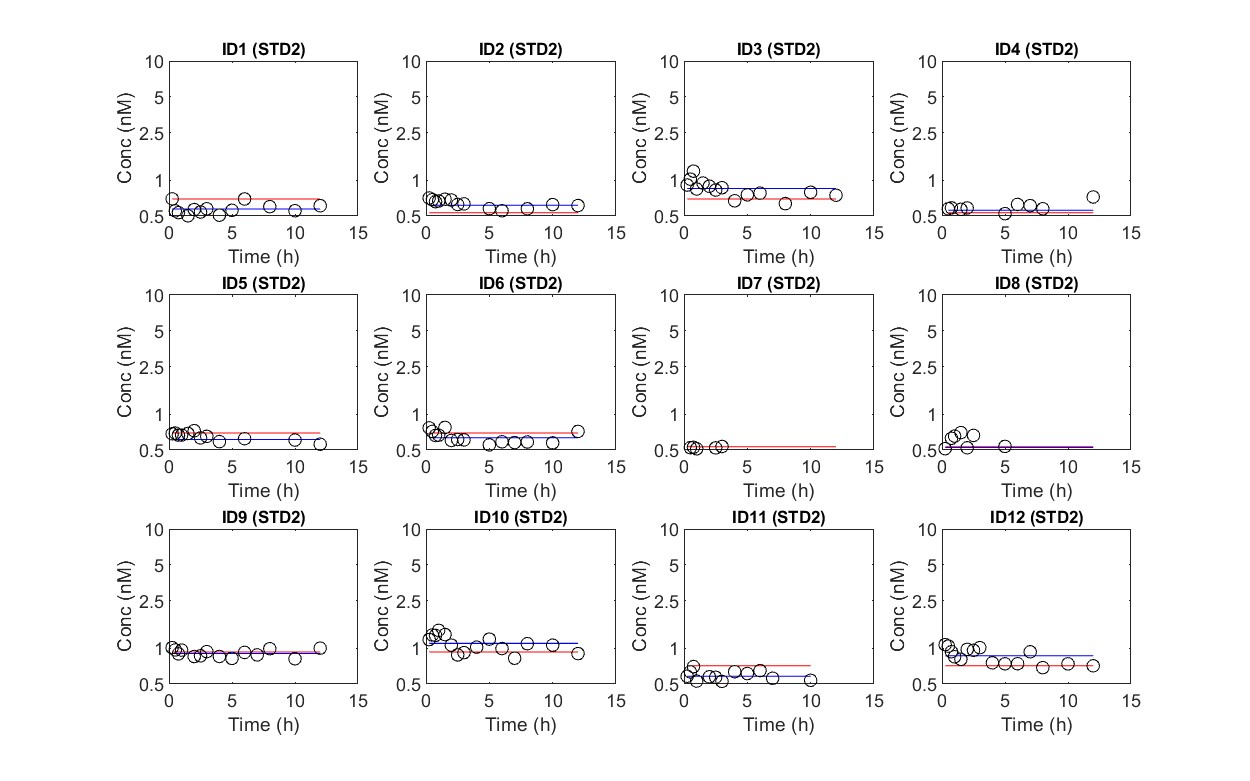

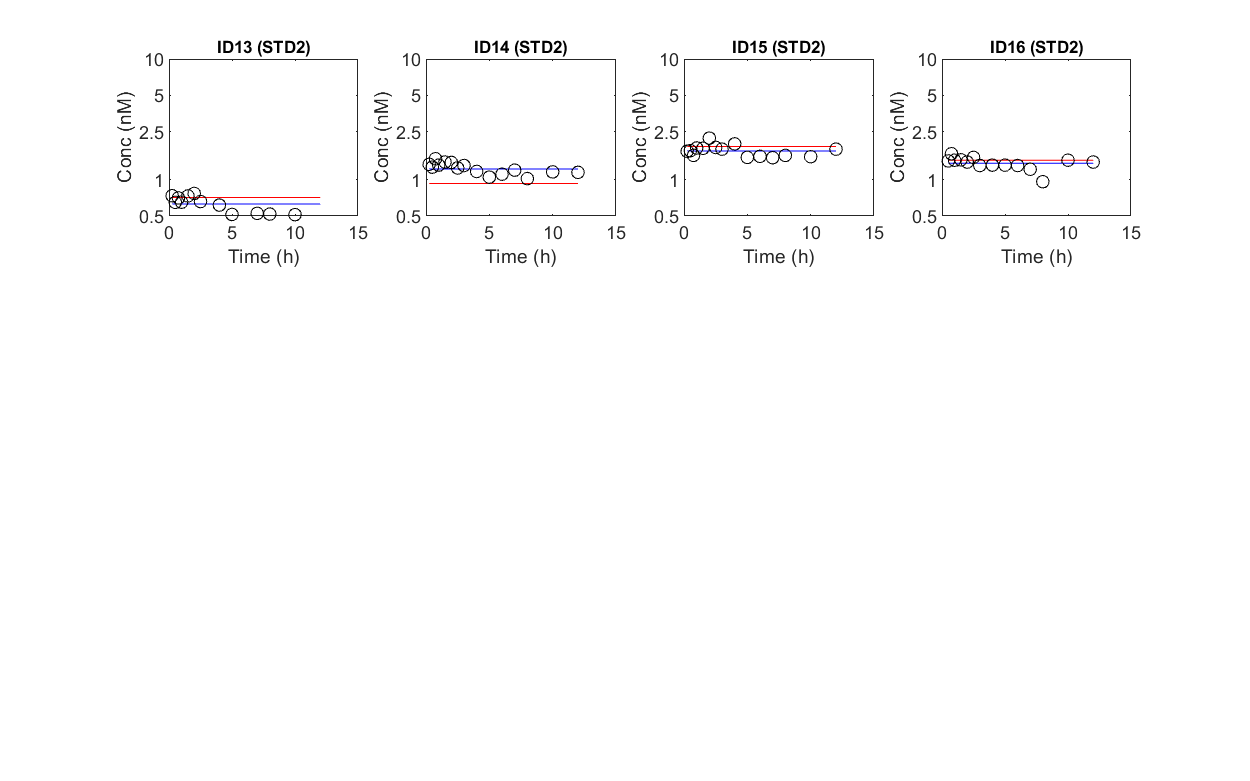

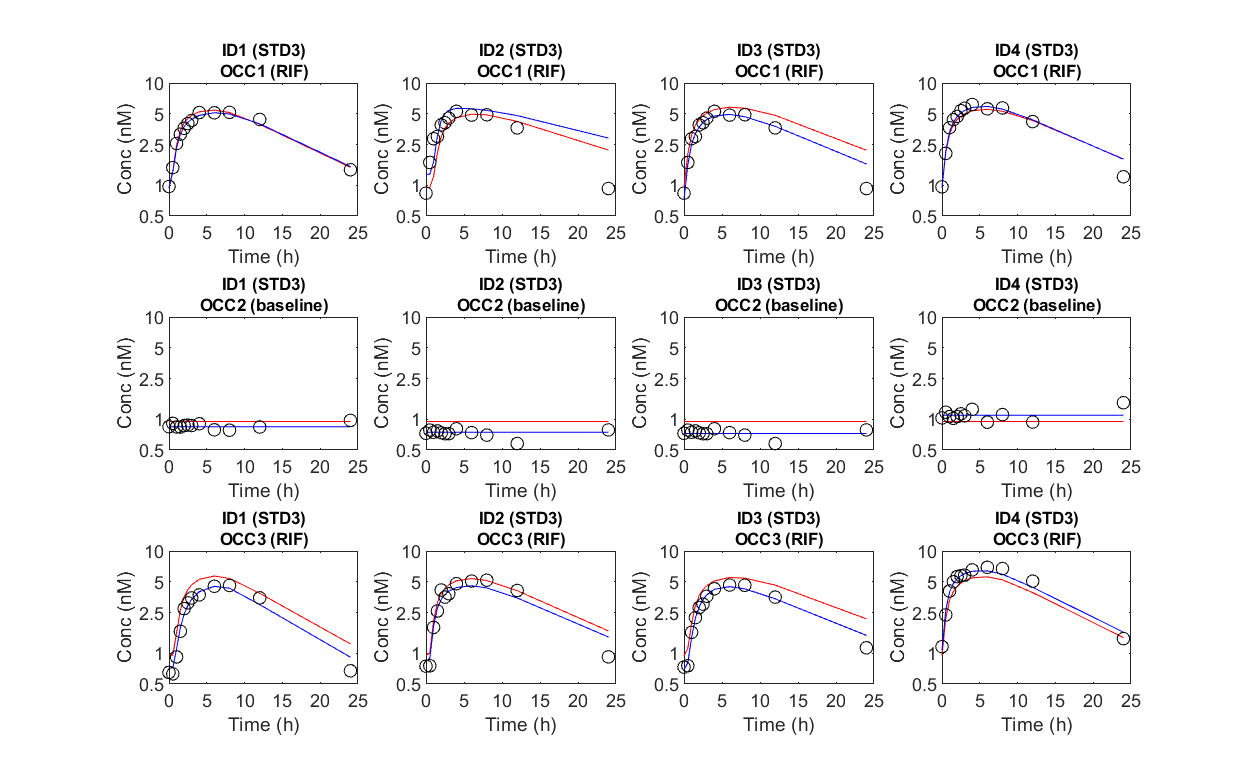

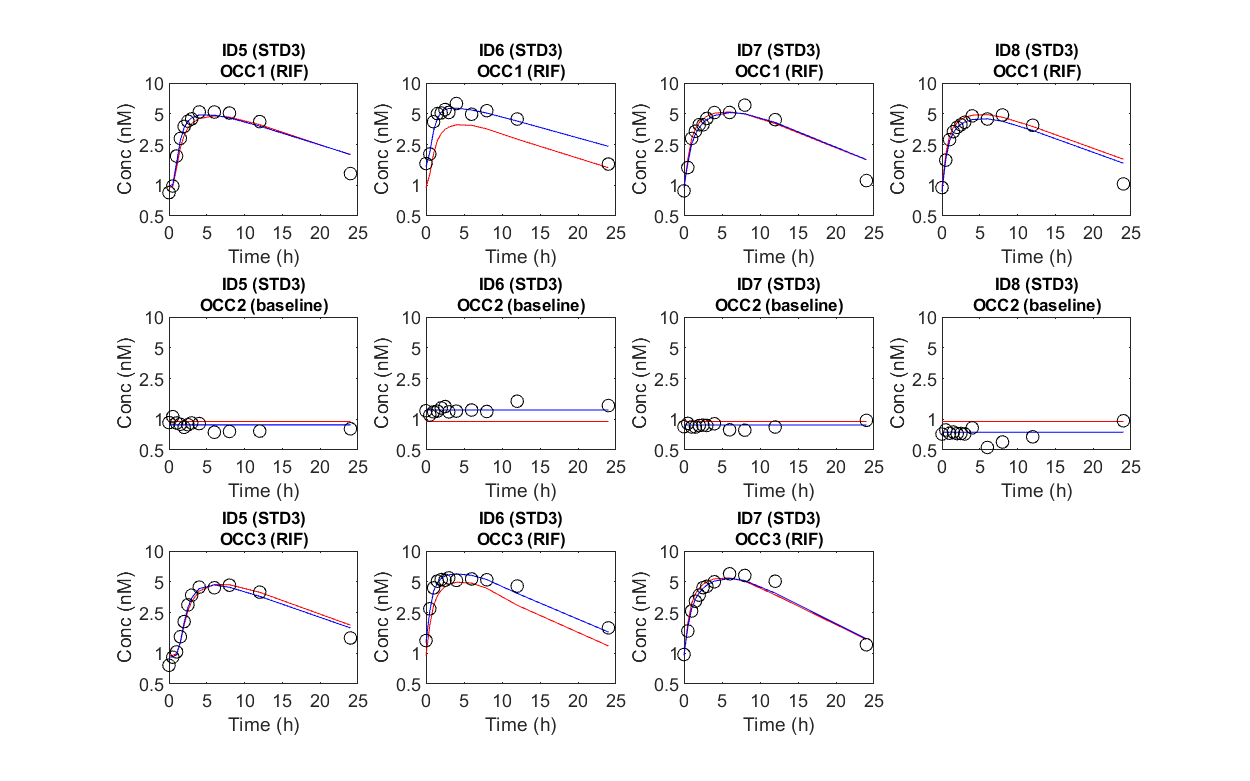

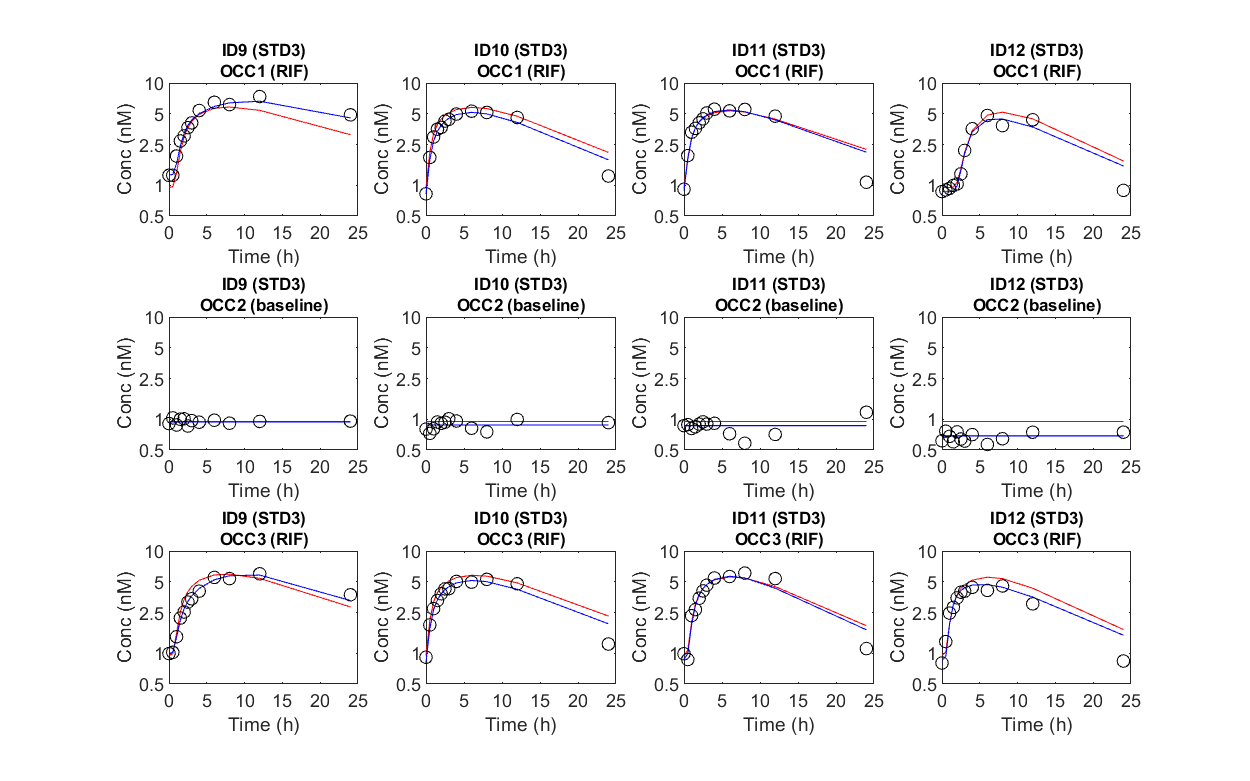


Individual CPI in urine


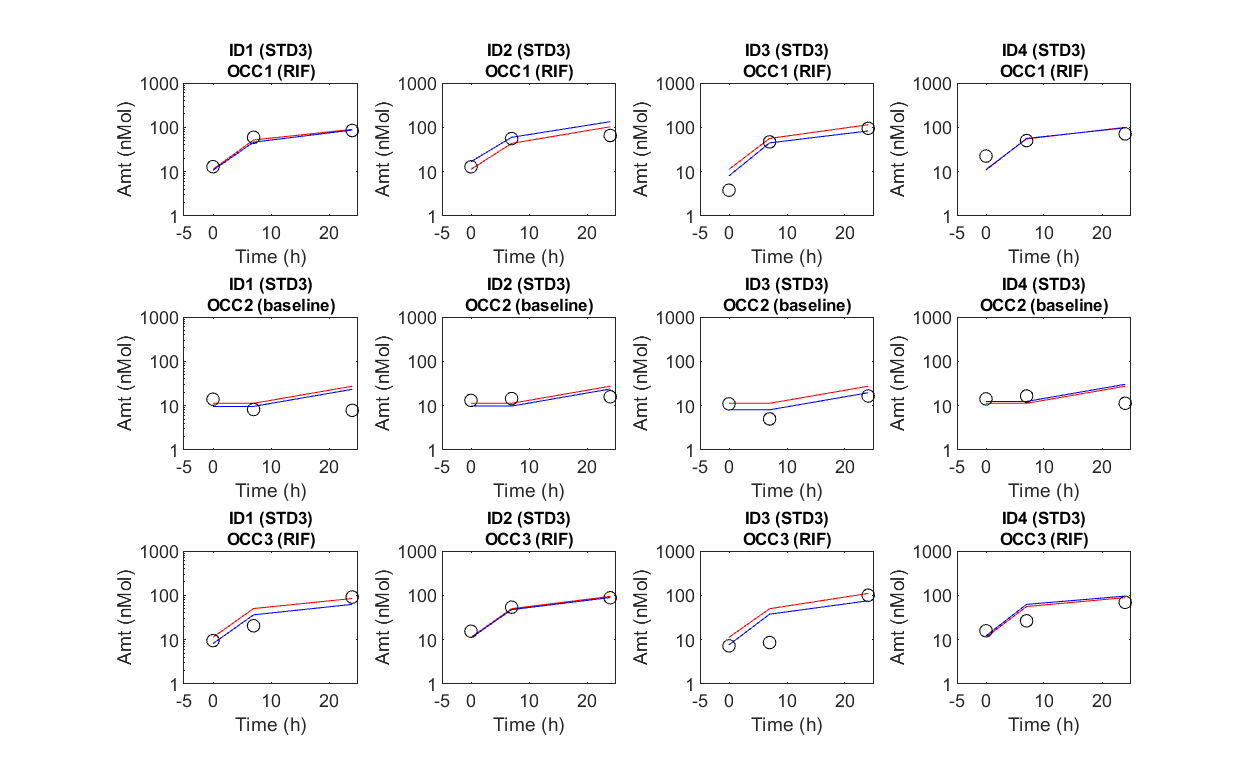

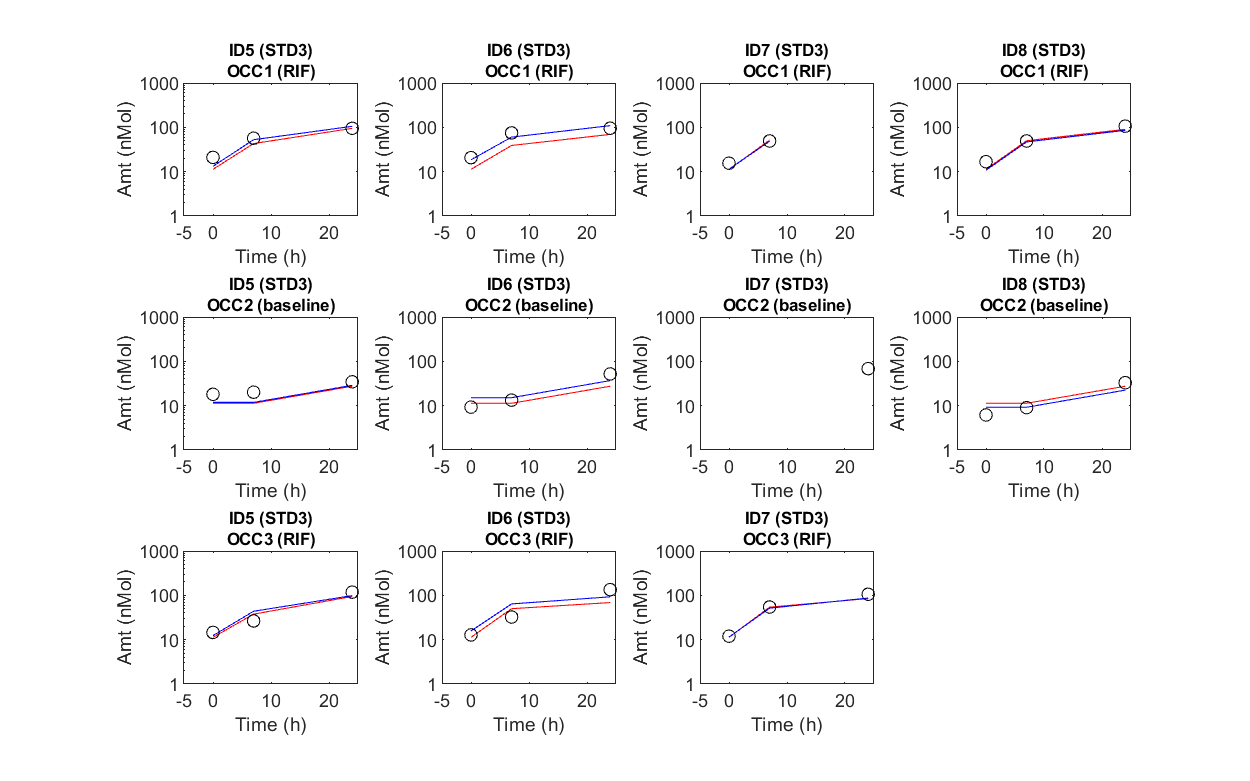

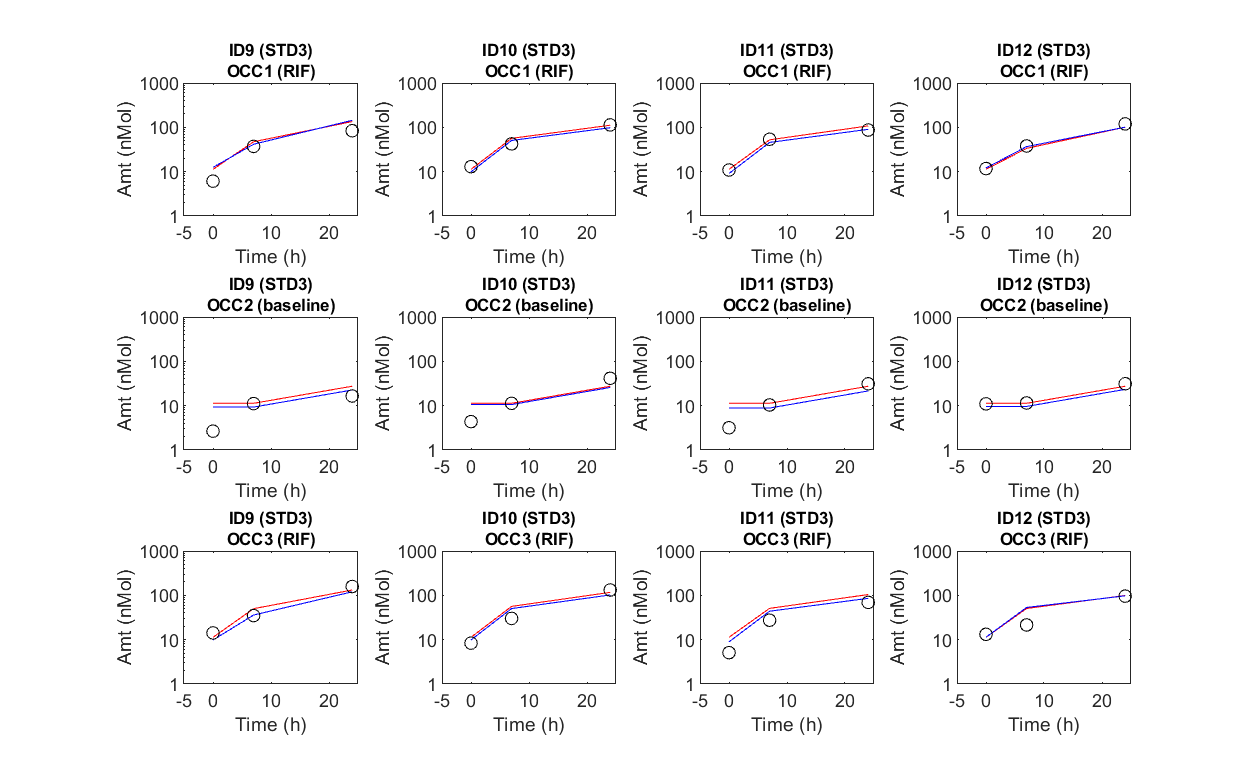


**Figure S5 Individual data for CPI in plasma and urine (black circles) and the fitted population (red line) and individual prediction (blue line) by the model**

Abbreviations: ID; identification number of each subject, STD; study, OCC; occasion, RIF; rifampicin.

# **Location of coproporphyrin I synthesis**

## **Biological evidence of the location of CPI synthesis**

A number of groups including us have reported CPI models with different assumptions about the location of CPI synthesis. Yoshikado et al. assumed CPI synthesis is in the liver based on the decreased urinary ratio of CPs (CPI and CP-III) to uroporphyrins (by-products produced from precursors of CPs, see **Figure S6**) in patients of the sporadic form of porphyria cutanea tarda (s-PCT), which shows decreased CPs synthesis in the liver and normal CPs synthesis in the erythrocyte^9,10^. On the other hand, Barnett et al. and Yoshida et al. assumed CPI synthesis in the blood^4,11^. Yoshida et al. discussed that the assumptions about the location of CPI synthesis had marginal impact on the parameter estimation, under the assumption that perpetrator drug does not influence intestinal re-absorption of CPI. In this study, a comprehensive literature search to gather evidence on the site of CPI synthesis was performed. The literature search showed that the evidence to support dominant CPI synthesis in the liver is inconclusive, although its partial contribution to total CPI in the body cannot be excluded, as discussed below. Yoshikado et al. assumed the dominant CPI synthesis in the liver based on the decreased urinary ratio of CPs to uroporphyrins in s-PCT^9,10^. However, this result in s-PCT patients should be interpreted carefully because the result in s-PCT patients does not necessary mean that CPI synthesis in the liver has large contribution to total CPI in the body. s-PCT patients showed significant accumulation of porphyrins in the liver (~30 times higher than normal controls) due to disrupted porphyrin homeostasis^10^, which does not necessary reflect the condition in healthy subjects. In addition, because urinary CPI consists relatively minor fraction of urinary CPs (30% of total CPs in healthy subjects^12^), the change in urinary CPs does not necessarily reflect the change in CPI disposition. Extrahepatic CPI synthesis is also suggested by the study that evaluated the fraction of radiolabelled CPs in urine after dosing radiolabelled delta-aminolevulinic acid (δ-ALA), a precursor of CPs synthesis^13^. Exogenously administered δ-ALA is preferentially incorporated into hepatic heme, and little is utilized for heme synthesis in the blood cells. Dosing radiolabelled δ-ALA resulted in the smaller fraction of radiolabelled CPI in urine (6% of CPs) relative to the composition of CPI in urine (30% of total CPs in healthy subjects^12^), meaning that CPI synthesised in the liver partially consists of urinary CPI, and the rest is accounted for by other routes of synthesis.

Further literature search identified evidences suggesting the substantial contribution of CPI synthesis in the blood to total CPI in the body. Several studies showed that urinal CPI excretion changed in parallel with erythropoiesis activity in the blood; blood loss conditions such as bleeding^14,15^, anaemia^16^, or haemolysis^17^ results in increased urinary CPI, accompanied by activated erythropoiesis as an adaptive response. In addition, substantial decrease in urinary CPI was observed when antineoplastic drugs suppressed erythropoiesis^13^. These evidences strongly suggest the blood cells are one of the main locations of CPI synthesis. However, because these studies lacked evaluation of the CPI synthesis in the liver, the contribution of hepatic CPI synthesis cannot be excluded.

In conclusion, the location of CPI synthesis was inconclusive based on the information available in the literature. Several studies supported substantial contribution of CPI synthesis in the blood to total CPI in the body, but partial contribution of hepatic CPI synthesis cannot be ruled out.


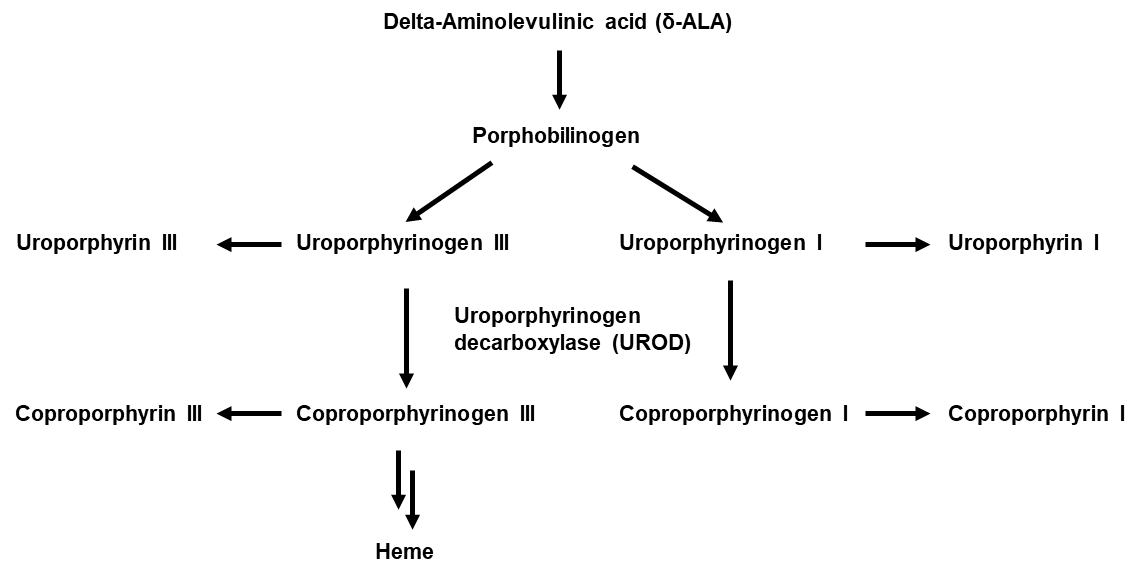


**Figure S6 Pathway of coproporphyrin I synthesis**

## **Parameter estimation in the model with CPI synthesis assumed to occur in the liver**


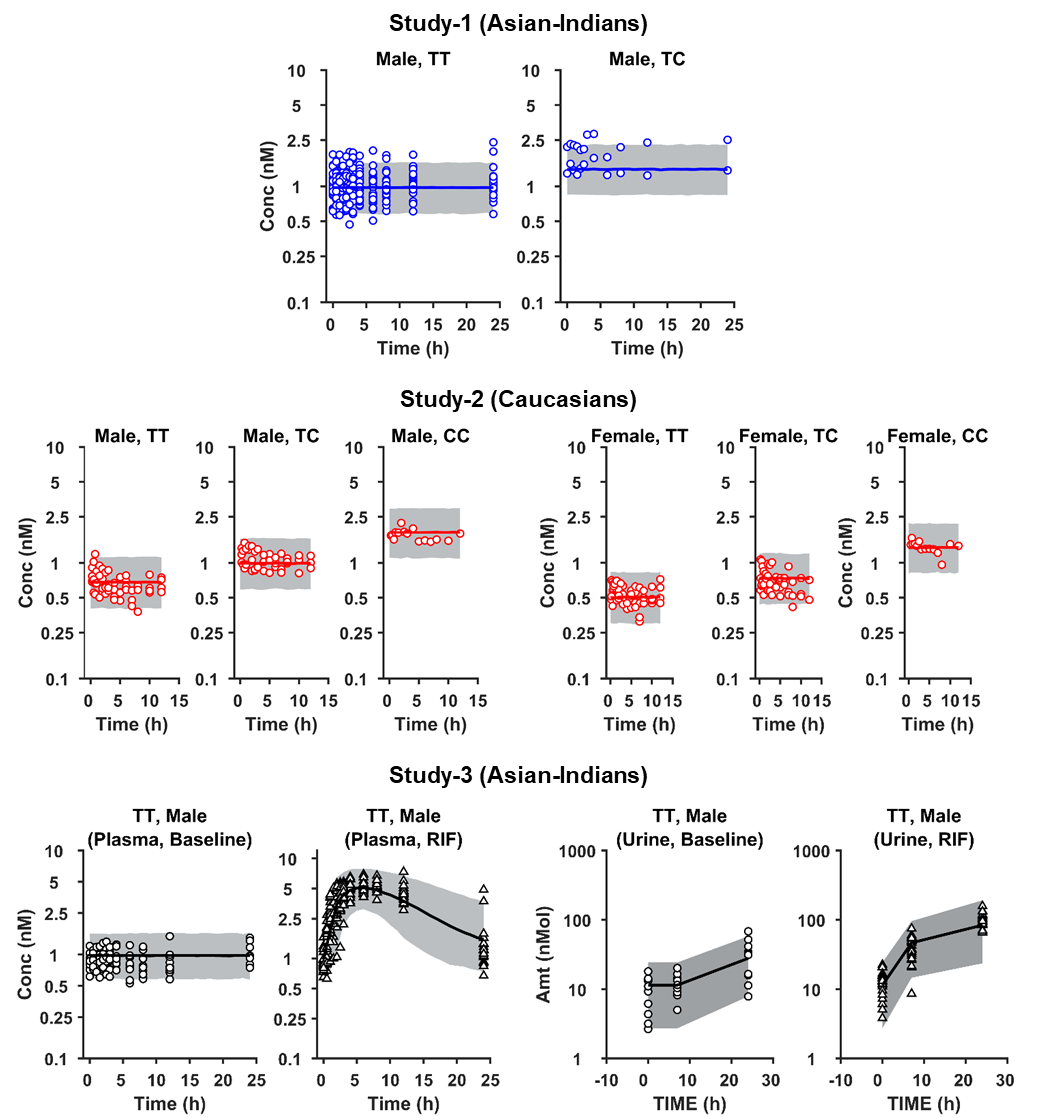


**Figure S7 Visual predictive check (VPC) of the mechanistic population PK model with CPI synthesis in the liver compartment**

Symbols, solid lines, and grey areas represent observed data, median population prediction, and 95% prediction intervals (n=5000), respectively. Simulations were performed for each sub-group including subjects with different sex or *SLCO1B1* c.521 (OATP1B1 transporter) genotype (521TT (TT), 521TC (TC), and 521CC (CC)) in three clinical studies. The CPI model with *k_syn_* in the liver compartment was used for the simulation.


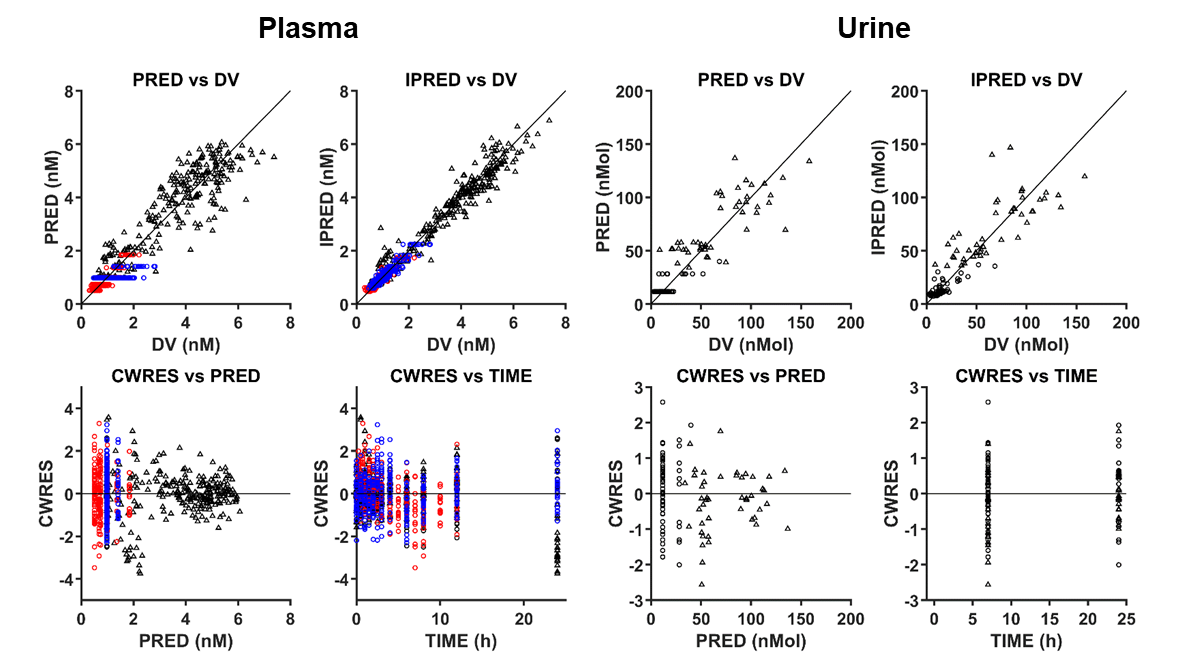


**Figure S8 Goodness-of-fit (GOF) plots for mechanistic population PK model with CPI synthesis in the liver compartment**

Abbreviations: DV; observed data, PRED; population prediction, IPRED; individual prediction, and CWRES; conditional weighted residuals. Colours represent clinical studies: blue - Study-1, red - Study-2, and black - Study-3. Circles and triangles represent occasions with or without rifampicin administration, respectively. Solid lines are reference lines. The CPI model with *k_syn_* in the liver compartment was used for the simulation.

## **Parameter estimations with different fraction of CPI synthesis in the liver compartment**


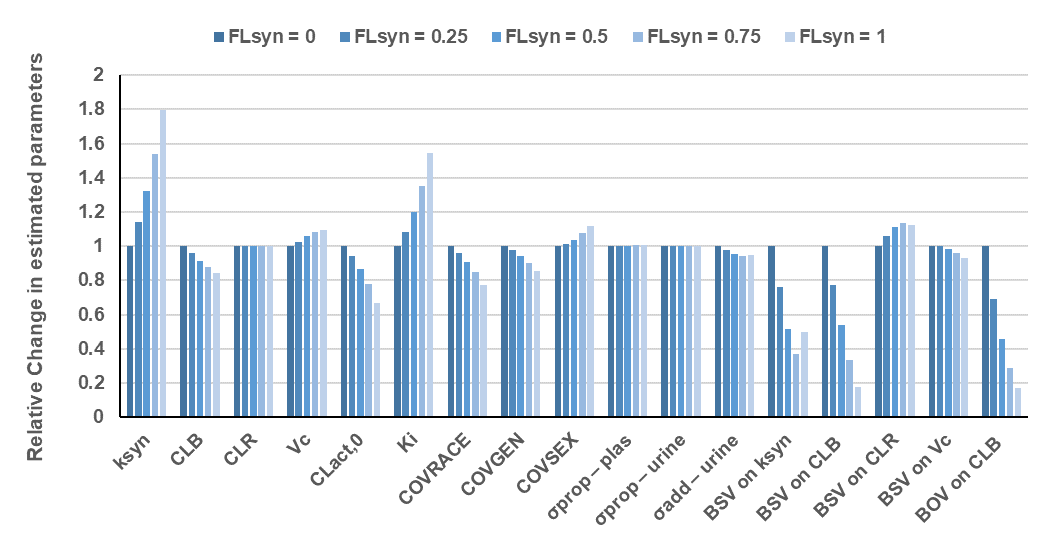


**Figure S9 Parameter estimations with different fraction of CPI synthesis in the liver compartment**

Relative change in estimated parameter values in models with different fraction of CPI synthesis in the liver compartment (*FL_syn_*) to the model with *FL_syn_* = 0 (CPI synthesis in the blood (central) compartment). Abbreviations; *k_syn_*; rate of CPI synthesis, *CL_B_*; biliary clearance, *CL_B_*; renal clearance, *V_c_*; volume of blood (central) compartment, *CL_uptake,0_*; hepatic active uptake clearance (*CL_active,u_*) in Caucasian male with *SLCO1B1* 521TT genotype, *K_i_*; inhibition constant of rifampicin for *CL_active,u_*, *FRAX*; surrogate variable of genetic effect, COV*_GEN_*; fractional change in *CL_active,u_* in *SLCO1B1* 521CC genotype, COV*_RACE_*; fractional change in *CL_active,u_* in Asian-Indians, *COV_sex_*; fractional change in *k_syn_* in female relative to male, σ_prop_; proportional residual error, σ_add_; additive residual error, BSV; between subject variability, BOV; between occasion variability.

# **Theoretical simulation of CPI-drug interaction in sub-populations**


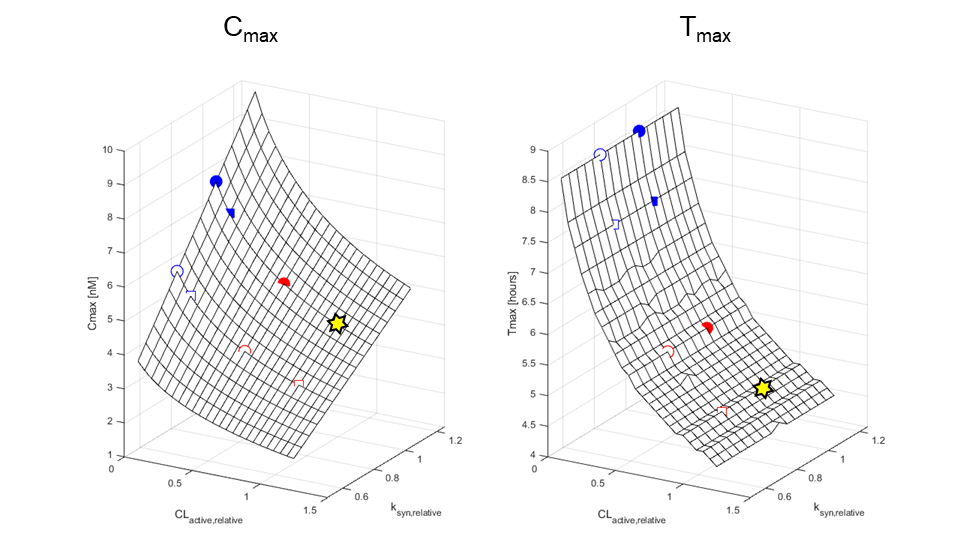


**Figure S10 Theoretical simulation of CPI-drug interaction across different CPI hepatic uptake clearance and synthesis rate.**

Simulation results of maximum concentration observed (C_max_) and time of maximum concentration observed (T_max_) of CPI caused by the theoretical rifampicin equivalent OATP1B inhibitor with different hepatic uptake clearance and endogenous synthesis rate, which were presented CL_active,u,relative_ and k_syn,relative_ as relative values to those in male Caucasians with 521TT (yellow star), respectively. Symbols on surface plots represent sub-populations simulated with the population (fixed effect) parameters of each sub-population; male (filled) and female (open), 521TT (red) and 521CC (blue), Caucasians (square) and Asian-Indians (circle).


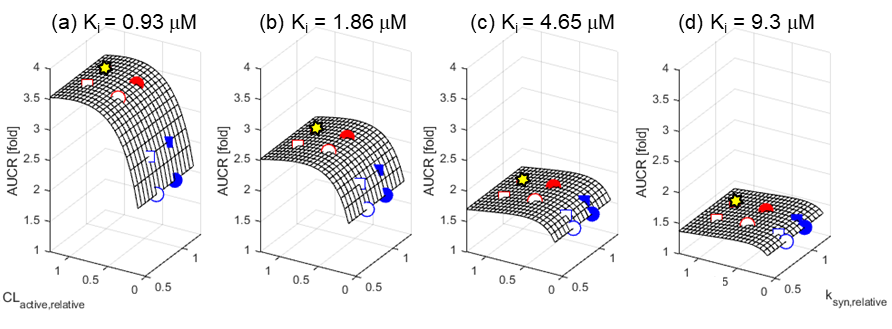


**Figure S11 Simulation of CPI-drug interaction by theoretical inhibitors with different *Ki***

Simulation of AUCR of CPI caused by theoretical OATP1B inhibitors with different total inhibition constants (*Ki*) (a) 0.93 μM, (b) 1.86 μM, (c) 4.65 μM, and (d) 9.3 μM. Simulations were performed with different combinations of hepatic uptake clearance and endogenous synthesis rate, which were presented *CL_active,u,relative_* and *k_syn,relative_* as relative values to those in male Caucasians with 521TT (yellow star), respectively. Symbols on surface plots represent sub-populations simulated with the population (fixed effect) parameters of each sub-population; male (filled) and female (open), 521TT (red) and 521CC (blue), Caucasians (square) and Asian-Indians (circle).

# References

1. Shen, H. *et al.* Evidence for the validity of pyridoxic acid (PDA) as a plasma-based endogenous probe for OAT1 and OAT3 function in healthy subjects. *J. Pharmacol. Exp. Ther.* **368**, 136–145 (2019).

2. Lai, Y. *et al.* Coproporphyrins in plasma and urine can be appropriate clinical biomarkers to recapitulate drug-drug interactions mediated by organic anion transporting polypeptide inhibition. *J. Pharmacol. Exp. Ther.* **358**, 397–404 (2016).

3. King-Ahmad, A. *et al.* A fully automated and validated human plasma LC-MS/MS assay for endogenous OATP biomarkers coproporphyrin-I and coproporphyrin-III. *Bioanalysis* **10**, 691–701 (2018).

4. Barnett, S. *et al.* Gaining Mechanistic Insight Into Coproporphyrin I as Endogenous Biomarker for OATP1B-Mediated Drug–Drug Interactions Using Population Pharmacokinetic Modeling and Simulation. *Clin. Pharmacol. Ther.* **104**, 564–574 (2018).

5. Ménochet, K., Kenworthy, K. E., Houston, J. B. & Galetin, A. Simultaneous assessment of uptake and metabolism in rat hepatocytes: A comprehensive mechanistic model. *J. Pharmacol. Exp. Ther.* **341**, 2–15 (2012).

6. Riccardi, K. *et al.* Comparison of species and cell-type differences in fraction unbound of liver tissues, hepatocytes, and cell lines. *Drug Metab. Dispos.* **46**, 415–421 (2018).

7. Kandoussi, H. *et al.* UHPLC-MS/MS bioanalysis of human plasma coproporphyrins as potential biomarkers for organic anion-transporting polypeptide-mediated drug interactions. *Bioanalysis* **10**, 633–644 (2018).

8. Valentin, J. & Streffer, C. Basic anatomical and physiological data for use in radiological protection: Reference values - ICRP Publication 89. *Ann. ICRP* **32**, 1–277 (2002).

9. Yoshikado, T. *et al.* PBPK Modeling of Coproporphyrin I as an Endogenous Biomarker for Drug Interactions Involving Inhibition of Hepatic OATP1B1 and OATP1B3. *CPT Pharmacometrics Syst. Pharmacol.* **7**, 739–747 (2018).

10. Moran, M. J. *et al.* Hepatic uroporphyrinogen decarboxylase activity in porphyria cutanea tarda patients: The influence of virus C infection. *Hepatology* **27**, 584–589 (1998).

11. Yoshida, K., Guo, C. & Sane, R. Quantitative Prediction of OATP-Mediated Drug-Drug Interactions With Model-Based Analysis of Endogenous Biomarker Kinetics. *CPT Pharmacometrics Syst. Pharmacol.* **7**, 517–524 (2018).

12. Benz-de Bretagne, I. *et al.* Urinary elimination of coproporphyrins is dependent on ABCC2 polymorphisms and represents a potential biomarker of MRP2 activity in humans. *J. Biomed. Biotechnol.* **2011**, 498757 (2011).

13. Shimizu, Y., Ida, S., Naruto, H. & Urata, G. Excretion of porphyrins in urine and bile after the administration of delta-aminolevulinic acid. *J. Lab. Clin. Med.* **92**, 795–802 (1978).

14. Dobriner, K. & Rhoads, C. P. THE EXCRETION OF COPROPORPHYRIN I FOLLOWING HEMORRHAGE IN DOGS. *J. Clin. Invest.* **17**, 105–108 (1938).

15. Schwartz, S. & Wikoff, H. M. THE RELATION OF ERYTHROCYTE COPROPORPHYRIN AND PROTOPORPHYRIN TO ERYTHROPOIESIS. *J Biol Chem* **194**, 563–573 (1952).

16. Watson, C. J. The erythrocyte coproporphyrin: Variation in Respect to Erythrocyte Protoporphyrin and Reticulocytes in Certain of the Anemias. *Arch. Intern. Med.* **86**, 797–809 (1950).

17. Dobriner, K. & Rhoads, C. P. THE PORPHYRINS IN HEALTH AND DISEASE. *Physiol. Rev.* **20**, 416–468 (1940).
